# Agropastoral and dietary practices of the northern Levant facing Late Holocene climate and environmental change: Isotopic analysis of plants, animals and humans from Bronze to Iron Age Tell Tweini

**DOI:** 10.1371/journal.pone.0301775

**Published:** 2024-06-12

**Authors:** Benjamin T. Fuller, Simone Riehl, Veerle Linseele, Elena Marinova, Bea De Cupere, Joachim Bretschneider, Michael P. Richards, Wim Van Neer

**Affiliations:** 1 Laboratory of Biodiversity and Evolutionary Genomics, Centre for Archaeological Sciences, University of Leuven, Leuven, Belgium; 2 Institute of Archaeological Science, University of Tübingen and Senckenberg Center for Human Evolution and Palaeoenvironment (HEP), Tübingen Germany; 3 Royal Belgian Institute of Natural Sciences, Brussels, Belgium; 4 Department of Archaeology, Ghent University, Ghent, Belgium; 5 Department of Archaeology, Simon Fraser University, Burnaby, British Columbia, Canada; New York State Museum, UNITED STATES

## Abstract

One of the largest isotopic datasets of the ancient Eastern Mediterranean region is evaluated, based on plants (n = 410), animals (n = 210) and humans (n = 16) from Tell Tweini (Syria). Diachronic analysis of plant and faunal specimens from four main periods of occupation: Early Bronze Age (2600–2000 BC), Middle Bronze Age (2000–1600 BC), Late Bronze Age (1600–1200 BC) and Iron Age (1200–333 BC) were investigated. Mean Δ^13^C results from seven plant species reveal emmer and free threshing wheat, olives, bitter vetch, rye grass and barley were adequately or well-watered during all periods of occupation. The grape Δ^13^C results suggest excellent growing conditions and particular care for its cultivation. The δ^15^N results indicate that especially the emmer and free threshing wheats received some manure inputs throughout the occupation sequence, while these were likely further increased during the Iron Age, encompassing also the olive groves and grape vineyards. Generally, domestic animals (cattle, sheep, goats) had C_3_ terrestrial diets and were kept together in similar environments. However, some animals consumed significant amounts of marine or C_4_ plants, possibly from disturbed habitats due to land use pressure or salt tolerant grasses and shrubs from wetland environments, which were recorded in the direct vicinity of the site. Middle Bronze Age humans consumed a C_3_ terrestrial diet with no measurable input from C_4_, freshwater or marine protein sources. Interestingly, the human diet was relatively low in animal protein and appears comparable to what is considered today a typical Mediterranean diet consisting of bread (wheat/barley), olives, grapes, pulses, dairy products and small amounts of meat. The combined isotopic analysis of plants, animals and humans from Tell Tweini represents unbroken links in the food chain which create unparalleled opportunities to enhance our current understanding of environmental conditions, climate change and lifeways in past populations from the Eastern Mediterranean.

## Introduction

**Fig 1:**
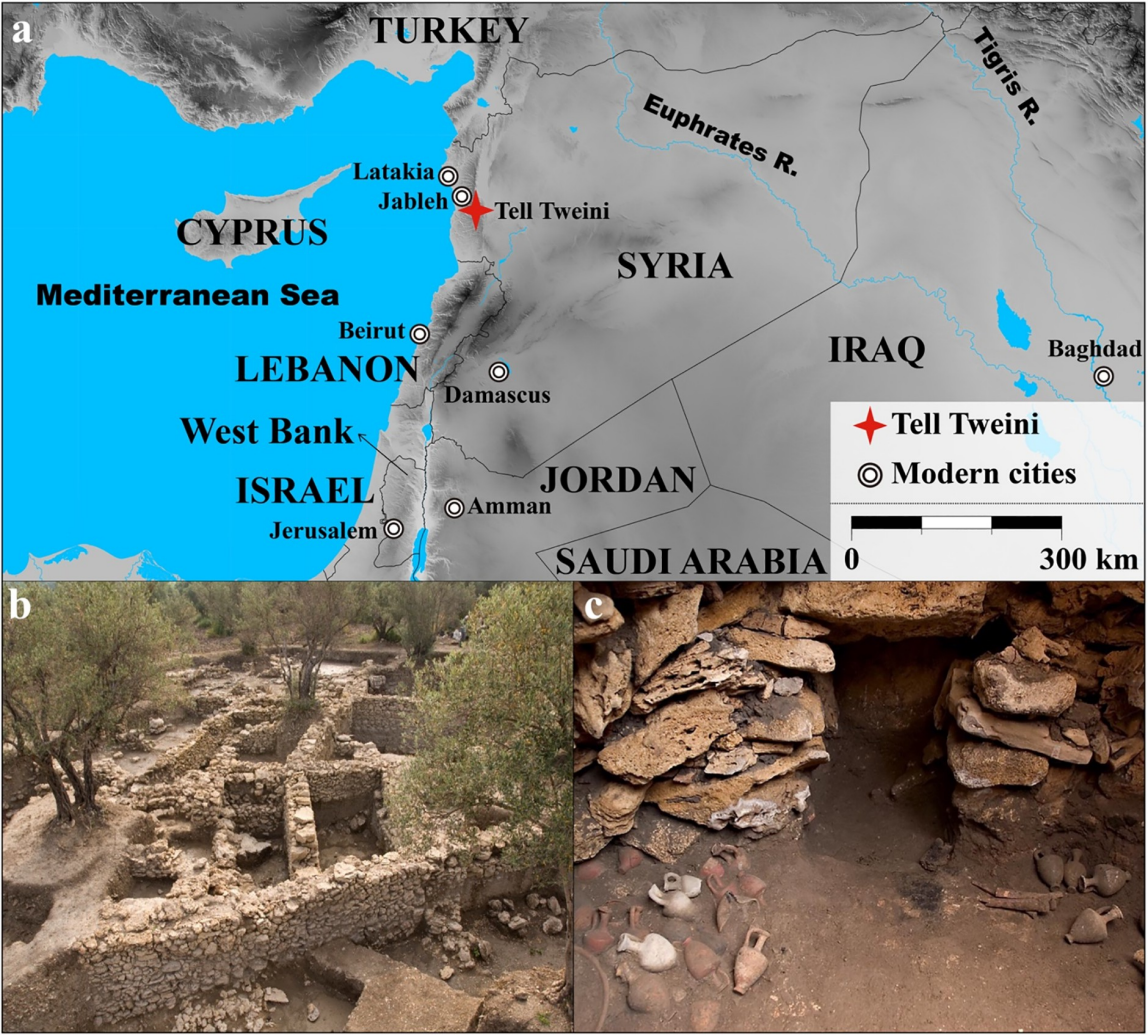
Map and photos of Tell Tweini. (a) Map of the Eastern Mediterranean showing the location of Tell Tweini in modern-day Syria. (b) Photo of Bronze and Iron Age houses from the Field A excavations (taken by Joachim Bretschneider). (c) Photo of Middle Bronze Age grave with Cypriote ceramics (taken by Joachim Bretschneider). (Map was generated using GMT 5.2.1. with the final layout created using Adobe Illustrator CC 2019 V.23.1.1.).

Plant, animal and human remains recovered from archaeological deposits represent invaluable resources to better understand the past. Locked within these specimens at the atomic level are stable isotope ratios of carbon (δ^13^C) and nitrogen (δ^15^N), which when measured, act as proxies to directly reconstruct a wealth of information related to agricultural practices, local climate patterns, animal husbandry and palaeodiet [1–6]. However, it has only been relatively recently that all three of these key resources have been simultaneously measured in archaeological sites to explore these topics in more detail (e.g., [7, 8]). Here a large-scale isotopic study focused on plants, animals and humans is presented for the site of Tell Tweini, Syria (Fig 1A–1C). Given the important historical significance of Tell Tweini, ancient Gibala, in the northern Levant, the goals of this research are to diachronically investigate agropastoral strategies and diet during the Bronze to Iron Ages of this major harbor site of the Ugaritic Kingdom.

The main specimens selected for isotopic analysis (plant seeds (n = 410), animal bones (n = 296)) date to four main periods of occupation: Early Bronze Age (2600–2000 BC), Middle Bronze Age (2000–1600 BC), Late Bronze Age (1600–1200 BC) and Iron Age (1200–333 BC). The selected human bones (n = 44) are from a Middle Bronze Age mass inhumation, enabling dietary reconstruction which is representative of a social group within a narrow time period. To date, only a limited number of isotopic studies have focused on Bronze and Iron Age archaeological sites from Syria (e.g., [9–16]). Given the large number of isotopic measurements and the extraordinary long-term continuity of the settlement, which includes the major climatic fluctuations at 4.2 and 3.2 kyr BP, this research allows an investigation into the effects of climate change on the society and the economy at Tell Tweini. Thus, this work provides a detailed and complete picture of agropastoral, environmental and palaeodietary practices of an Eastern Mediterranean society and how these varied during different phases of occupation throughout the Bronze and Iron Age.

### Archaeological and environmental context of Tell Tweini

Tell Tweini (35°22′17.93″N, 35°56′12.60″E) is an archaeological site located at an elevation of 19–27 meters above sea level and covering a surface area of 11.6 ha [17]. It is situated at the confluence of two rivers (Nahr ar-Rumailah and Nahr al-Fawwar) near the modern town of Jableh and about 30 km south of the modern city of Lattakia on the Syrian coast. Tell Tweini can likely be identified with the ancient city of Gibala, the southernmost town of the Ugarit Kingdom [18], and one of its four harbors (Late Bronze Age, ca. 1600–1200 BC). Excavations at Tell Tweini yielded materials from the Early Bronze Age (ca. 2600–2000 BC) but only a few of the excavated loci were dated to this period and were largely disturbed by superjacent features. The main period of occupation was between the Middle Bronze Age and Iron Age I/II (2000–700 BC). During the Middle Bronze Age (ca. 2000–1600 BC), several small states emerged in the Levant which led to the development of complex commercial trade networks [19]. The Middle Bronze Age I (ca. 2000–1800 BC) is an enigmatic period in the area of Tell Tweini, with only a few settlements and tombs present and a likely reduced settlement [20]. Most of the intramural tombs excavated at Tell Tweini date to Middle Bronze Age II (ca. 1800–1600 BC). The Late Bronze Age (ca. 1600–1200 BC) represents a period of prosperity in the economic and cultural history of Syria and of the entire Eastern Mediterranean. It was the time of the great Ugarit Kingdom, of which ancient Gibala/Tell Tweini was a part until the fall of the Ugarit Kingdom shortly after 1200 BC. The many imported luxury goods found at the site confirm the existence of an elaborate network of international relations, and resource transfer especially with Cyprus, Crete, Syro-Mesopotamia, the Hittite Empire and Egypt [17]. The final Late Bronze Age settlement saw some destruction but Tell Tweini continued, no longer part of the Ugarit Kingdom, in the Iron Age I, II and III until roughly 500 BC.

Based on the well-investigated ‘Rapid Climate Change’ events at 4.2 and 3.2 ka BP [21–26], various climate-related conclusions have been drawn about ancient agricultural practices [27]. Sustainable landscape features around Tell Tweini were described as a wetland characterized by dense reeds, shallow pools, and a fluctuating groundwater table at around 7500–7000 cal BP [28]. Fluvial conditions and vegetation cover were fluctuating, but the spring source of the Al-Fawar rivulet is still located south of the site and the swampy marsh vegetation in the wetland formed ideal conditions for the genesis of tufa. Peat accumulations are evident at ca. 4000 cal BP which agrees with the pollen data indicating moister conditions from 3900 to 3500 cal BP [27]. The wetland persisted between 4000–3000 BP through moisture input from local springs during a climatically extended drier period, characterized around Tell Tweini by xerophytic woods and shrubs [27], also termed ‘Cold Period 4’ [29] and created a contrasting landscape of dry vegetation elements and local wetland spots around the site. The sequence of increased climatic aridity includes the Tell Tweini settlement phases XB and beginning of IXA, i.e., the Early Bronze Age IVB and beginning of the Middle Bronze Age I, which is characterized by reduced occupation at the site. The subsequent Middle Bronze Age II settlement (phase IXB) has been described as the revival of urbanization [30].

The 3.2 kyr cal BP drying event [31, 32], lasting until the 9^th^ century BC has been related to migration and political and economic instability in the wider region [33]. The destruction of several coastal sites including Ugarit and Tell Tweini in the first quarter of the 12^th^ century was linked to maritime and land migrations of the ‘Sea Peoples’ which was one of several factors that might have caused the collapse of the Late Bronze Age socioeconomic system [34, 35]. The first post-destruction Iron Age settlements in the northern Levant, including Tell Tweini, included poor dwellings in the ruins of the former prosperous Late Bronze Age towns where local ceramic traditions but also new forms of military technology from Italy and the Aegean came into play.

While the wetland around Tell Tweini still persisted throughout the Late Bronze Age, it is assumed that climatic aridity was an issue from the final Late Bronze Age II on, but especially during the Early Iron Age I (1200–900 BC). This time sequence is associated with a ‘warm steppe/hot desert biome’ [27] which is also termed “Cold Period 3” [29], and thus agricultural practices and the economy would have required adaptive measures [20]. The whole Cold Period 3 sequence includes the collapse of the Ugarit Kingdom shortly after 1200 BC, and the later Iron Age I destruction level at Tell Tweini (phase VIIB), which is also marked by flash precipitation [28], as well as the earlier part of state formation in the Iron Age II (Tweini VI A-B).

Other than no longer being part of the Ugarit Kingdom, Tell Tweini persisted throughout the 12^th^ and 11^th^ century BC likely with a changed organization of the community, as suggested by different habitation features [36]. Over the course of the 11^th^ century BC, Tell Tweini developed into a new regional center with the revived exchange (of goods and people) with Cyprus [37] After a second destruction by fire at the end of the 11^th^ century, the site was completely redeveloped by the start of the 9^th^ century BC. Increased occupation is noted for the beginning of Iron Age II directly on top of the Iron Age I city with several buildings of the Iron Age I being re-used. This renewal of urban culture on the Levantine coast can be connected to the new trade networks between Cyprus and inner Syria. Again, numerous imported Cypriot pots were found at Tell Tweini. However, the environmental scenario at Tell Tweini is puzzling since the renewal of urban culture coincides with the full development of desert conditions at ca. 900 BC, a process that started shortly after 1200 BC [27]. Baeteman and Bogemans [28] recognized in the geoarchaeological record short shifts to wetter conditions between ca. 1000–950 BC, which they explain with the occurrence of flash precipitation. It is possible these storms sufficed to fill the groundwater reservoir which was needed for ongoing agricultural production around the site.

At the beginning of the 8^th^ century BC, the Syrian coast came under Assyrian control. The trade towns kept a certain amount of autonomy but had to pay large tributes, and at the end of the 8^th^ century, another phase of architectural renewal took place. A well-preserved plan of the city during the Iron II/III period (ca. 900–500 BC) existed. Production of olive oil and wine were considered a main economic activity as suggested by findings of oil presses and refining installations in many houses [20]. While much has been learned about Tell Tweini through archaeological excavation and historical sources [38], isotopic measurements can provide additional insights into the conditions of agricultural production, animal management and dietary habits of the people which can enhance our current understanding of the cultural history of the Eastern Mediterranean.

## Materials and methods

### Sample selection

From 1999 to 2010, a Belgian and Syrian team of researchers worked at Tell Tweini (see Bretschneider and Van Lerberghe [39] and Bretschneider and Jans [17] for the results of the Belgian team). The archaeobotanical data used for reconstructing the plant economy at Tell Tweini were collected during the excavation seasons 1999–2010. The data is based on 7918 identifications of charred plant remains from 76 soil samples with a total volume of 1605 liters. The plant materials were extracted from the sediment by machine floatation and identified using a stereomicroscope magnification (6-50x) and modern reference collections. In addition, illustrated atlases [40, 41], identification guides [42] and further publications [43] were used for identification. Intact and undistorted charred grains or seeds or fruit stones were selected for isotopic analysis. For this study, the following species were studied: emmer wheat (*Triticum dicoccum*, n = 109), free threshing wheat (*Triticum aestivum/durum*, n = 53), grape (*Vitis vinifera*, n = 65), olive (*Olea europaea*, n = 87), the pulse bitter vetch (*Vicia ervilia*, n = 33), the wild ryegrass weed (*Lolium* cf. *multiflorum*, n = 30) and barley (*Hordeum vulgare*, n = 33).

Archaeozoological studies of the Belgian excavations at Tell Tweini (from 2006–2010) analyzed >45,000 vertebrate remains. Most of these remains were collected through hand-picking during the excavations, although part was also retrieved from the heavy residue of the floatation for botanical remains (1 mm mesh). Details on the methods of analysis can be found in Linseele et al. [44]. A subset of the species, representative of the different periods of occupation were selected for isotopic analysis, but some species were only represented by a small number of bones. The majority of the animal remains were found in fill layers from domestic settlement contexts. Dating of the faunal remains was based on the architectural stratification and associated ceramic assemblages of the site [39, 45]. For the purpose of this paper, the faunal remains were grouped by the larger periods, i.e., Early Bronze Age (ca. 2600–2000 BC), Middle Bronze Age (ca. 2000–1600 BC), Late Bronze Age (ca. 1600–1200 BC) and Iron Age (ca. 1200–333 BC), in order to have samples of sufficient size. The Iron Age material is predominantly Iron Age I/II (1200–700 BC). The fauna selected for analysis include: dog (*Canis lupus* f. familiaris, n = 20), cattle (*Bos primigenius* f. taurus, n = 63), sheep (*Ovis ammon* f. aries, n = 56), goat (*Capra aegagrus* f. hircus, n = 56), suids (domestic pig *Sus scrofa* f. domestica and wild boar *Sus scrofa*, n = 26), Mesopotamian fallow deer (*Dama mesopotamica*, n = 23), brown hare (*Lepus europaeus*, n = 5), brown bear (*Ursus arctos*, n = 3), gazelle (*Gazella subgutturosa*, n = 5), accipitrid birds of prey (n = 2), black kite (*Milvus migrans*, n = 1), buzzard (*Buteo buteo*, n = 2), chukar partridge (*Alectoris chukar*, n = 5), crows (*Corvus* sp., n = 4), ducks (Anatinae, n = 5), geese (*Anser* sp., n = 15), herons (*Ardea* sp., n = 1) and pigeons (*Columba* sp., n = 4). Fish remains recovered at Tell Tweini were previously analyzed and published in Fuller et al. [15].

The human bones were previously studied by Ricaut [46]. Most individuals were from a communal grave found under a house floor, and these individuals date to approximately 1700 BC based on associated finds such as large quantities of Cypriot pottery (Fig 1C) [46–48]. From this tomb, we selected all 34 right talus bones. These elements are all from adult individuals, but a sex identification is not possible on this bone. However, anthropological analysis of the other human bones found in the grave show that male and female individuals were equally represented. A total of 10 individuals could be selected from the other scattered graves, including one female and one male individual, as well as some children. The bones from the Middle Bronze Age communal grave are stored alongside other human remains from the Bronze and Iron Age in the Archaeological Depot of the Tell Tweini Excavation in Jebleh, Syria (35°22’15" N—35°56’16" E). Additional information regarding the ethical, cultural, and scientific considerations specific to inclusivity in global research is included in the S1 Checklist.

### Isotopic analysis

Bones were prepared for collagen extraction at the former Department of Human Evolution, Max Planck Institute for Evolutionary Anthropology in Leipzig, Germany, using the protocol outlined in Richards and Hedges [49], modified to include a final stage of ultrafiltration prior to lyophilization as described in Brown et al. [50]. Charred plant remains were also prepared in Leipzig as well as at the Institute of Geosciences at the University of Tübingen, Germany, following Vaiglova et al. [51], but without FTIR since the specimens were well preserved. To achieve optimal mass for carbon and nitrogen isotopic analysis, ~4–6 individual grains/seeds were crushed and homogenized and directly transferred to tin capsules. However, for the barley individual grains were isotopically measured. In all cases, well-developed grains/seeds were selected to guarantee the exclusion of isotopic values that would not reflect the mean growing conditions, e.g., such as values from immature grains. All plants were analyzed for δ^13^C and δ^15^N results except for the barley for which only δ^13^C measurements were obtained.

Isotopic measurements were carried out at the Institute of Geosciences (University of Tübingen) on a FinniganMAT252 gas source mass spectrometer with a ThermoFinnigan GasBench II/CTC Combi-Pal autosampler. Prior to mass-spectrometric measurements, the barley grains were reacted with 5% HCl to eliminate sedimentary carbonate. In Leipzig, specimens were combusted to CO_2_ and N_2_ in an automated carbon and nitrogen analyzer (Carlo Erba) and analyzed for stable isotope ratios with a continuous-flow isotope ratio-monitoring mass spectrometry (PDZ Europa Geo 20/20). The stable isotopes were measured as the ratio of the heavier isotope to the lighter isotope (^13^C/^12^C or ^15^N/^14^N) and reported as δ values in parts per 1,000 or ‘‘per mil” (‰) relative to internationally defined standards for carbon (Vienna Pee Dee Belemnite, VPDB) and nitrogen (Ambient Inhalable Reservoir, AIR) [52]. All samples were measured against the IAEA N1, N2, CH6 and CH7 isotope standards as well as two internal standards (bovine liver and methionine) and errors were calculated using the average variation of the internal standards over at least one year of measurements and were less than 0.2‰ for both δ^13^C and δ^15^N.

Plant δ^13^C values, a basic component of the fixation of carbon during photosynthesis, mainly reflects drought stress during the grain-filling period of a plant, due to closure of the stomata to avoid dehydration through increased evapotranspiration, thus leading to increased use of ^13^C in the photosynthetic cycle [53]. Plant δ^15^N values basically reflect the nitrogen sources within the soil and can be used to reconstruct ancient manure input [53]. Plant δ^13^C and δ^15^N results were corrected by subtracting 0.1‰ and 0.3‰, respectively, to account for the minor effects of charring [54]. To compare plant isotopic results from different time periods, fluctuations in atmospheric CO_2_ concentrations (δ^13^C_air_) over time need to be considered. To account for this, the Tell Tweini data was also calibrated into Δ^13^C values using the AIRCO2_LOESS data application [55, 56]. This application is based on CO_2_ captured in the Antarctic ice core and the inbuilt formula by Graham Farquhar. For statistical analysis Shapiro-Wilk tests were employed first to verify the normality of the datasets. For datasets that followed a normal distribution (Shapiro-Wilk P value >0.05), a Levene`s test and an Independent-samples t-test were then employed to assess the homogeneity of variances of the datasets and to determine if the means of the analyzed datasets were significantly different from each other. For datasets that rejected the assumption of normal distribution (Shapiro-Wilk P value <0.05), the nonparametric Mann-Whitney U Test was employed. The statistical analyses were performed using the SPSS Statistics 26 package and complete statistical information for the plants and animals are listed in S1 and S2 Tables in S1 Data.

## Results

The following tables list the information and isotopic results for the plants (S3 Table in S1 Data), animals (S4 Table in S1 Data) and humans (S5 Table in S1 Data). Unfortunately, a large number of the animal and human specimens failed to yield collagen. In addition, although some samples produced collagen with acceptable C:N results [57], they had low %C (< 13%) and %N (< 4.8%) values which resulted in their exclusion [58]. Thus, only 210 out of 296 (71%) animals and 16 out of 44 (36%) humans yielded acceptable isotopic results. The high failure rate of the collagen extraction at Tell Tweini is attributed to the hot and arid climate of the region which results in the degradation and poor survivability of organic molecules [59], and this was also encountered with the fish specimens that were previously isotopically analyzed [15].

### Early Bronze Age III-IV (ca. 2600–2000 BC)

**Fig 2:**
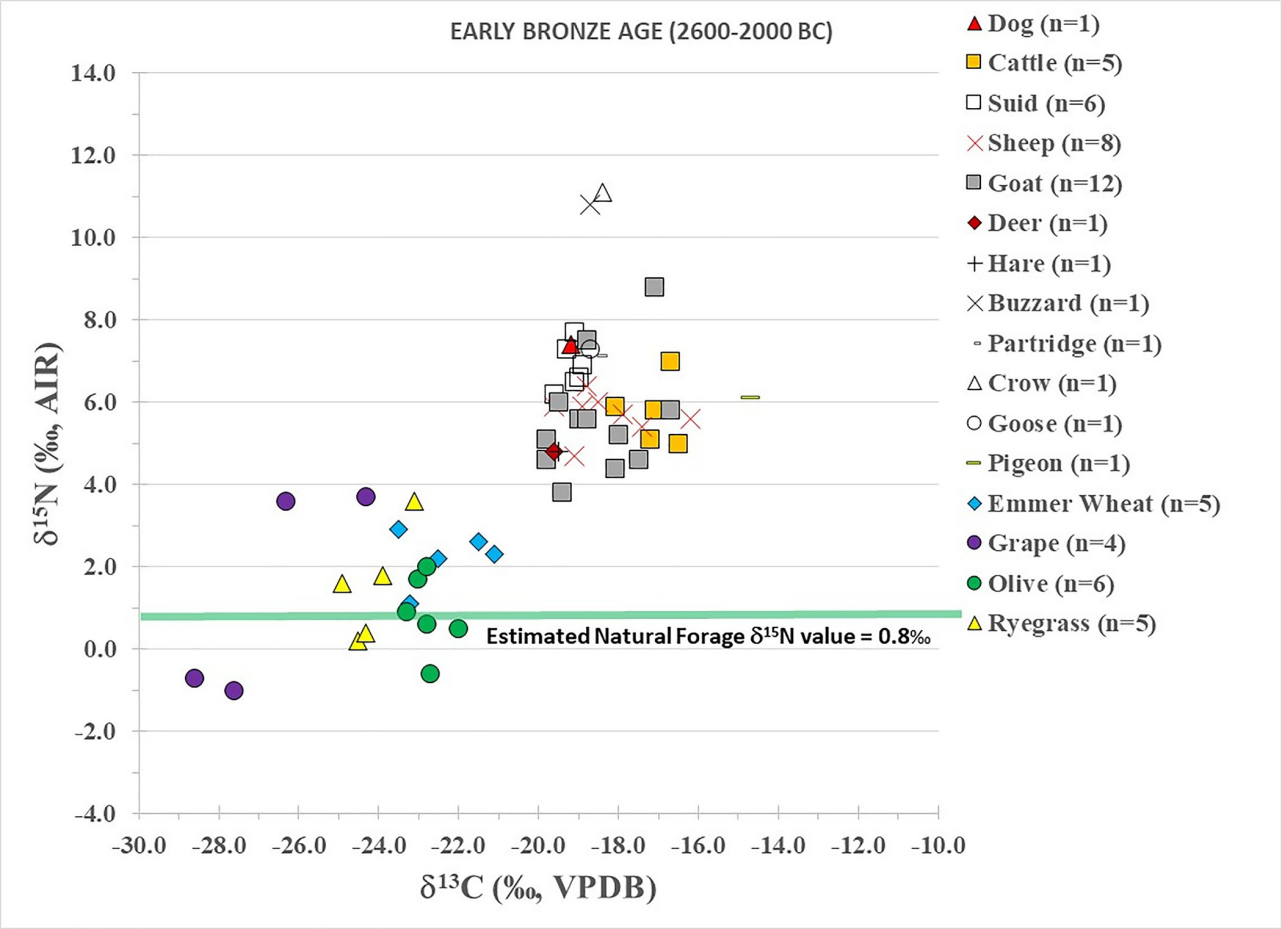
Early Bronze Age plant and animal isotopic results from Tell Tweini. Estimated natural forage δ^15^N value calculated by subtracting 4‰ from the average δ^15^N values of the deer and hare [8].

Early Bronze Age plant results are summarized in Table 1 and individually plotted in Fig 2 (except for the barley). The mean ± SD δ^13^C values are variable but the emmer wheat (-22.3±1.0‰), olives (-22.4±0.4‰), ryegrass (-24.0±0.7‰) and barley (-22.9±0.9‰) are similar, while the grapes (-26.6±1.9‰) are the most ^13^C-depleted. In contrast, the plants have mean ± SD δ^15^N values that are similar to each other, between 1–3‰: emmer wheat (2.5±0.7‰); olives (1.2±0.9‰); ryegrass (1.8±1.4‰); grapes (1.7±2.6‰).

**Table 1:** Summary of the mean ± SD isotopic results of the plants by time period from Tell Tweini. For the time periods and total columns, the first line = δ^13^C (mean ± SD); second line = δ^15^N (mean ± SD); third line = number of samples (n).

Plant	Early Bronze Age	Middle Bronze Age	Late Bronze Age	Iron Age	Total
**Emmer Wheat** ***(*** *Triticum dicoccum)*	-22.3±1.0‰ 2.5±0.7‰ n = 5	-22.8±0.7‰ 3.7±1.4‰ n = 39	-22.5±1.1‰ 3.3±1.0‰ n = 30	-22.6±1.0‰ 3.1±1.1‰ n = 35	**-22.6±0.9‰** **3.3±1.2‰** **n = 109**
**Free Threshing Wheat** **(***Triticum aest*.*/durum)*	-	-22.0±1.6‰ 3.5±1.2‰ n = 10	-22.6±0.7‰ 2.6±1.1‰ n = 23	-22.6±0.8‰ 3.8±1.7‰ n = 20	**-22.5±0.9‰** **3.2±1.4‰** **n = 53**
**Grape** *(Vitis vinifera)*	-26.6±1.9‰ 1.7±2.6‰ n = 4	-26.5±1.4‰ 1.6±1.3‰ n = 23	-26.6±0.9‰ 1.7±1.4‰ n = 25	-25.4±1.0‰ 3.1±2.3‰ n = 13	**-26.3±1.2‰** **1.9±1.7‰** **n = 65**
**Olive** *(Olea europaea)*	-22.4±0.4‰ 1.2±0.9‰ n = 6	-22.7±1.2‰ 1.8±.2.0‰ n = 29	-22.7±1.4‰ 1.3±.2.1‰ n = 28	-22.7±0.9‰ 2.1±.2.0‰ n = 24	**-22.7±1.1‰** **1.7±2.0‰** **n = 87**
**Ryegrass** *(Lolium cf*. *multiflorum)*	-24.0±0.7‰ 1.8±1.4‰ n = 5	-23.8±0.6‰ 3.0±.0.9‰ n = 10	-22.7±1.1‰ 2.2±.1.1‰ n = 10	-22.9±0.9‰ 3.1±.0.6‰ n = 5	**-23.3±1.0‰** **2.5±1.1‰** **n = 30**
**Bitter Vetch** *(Vicia ervilia)*	-	-23.6±1.2‰ 1.2±.0.4‰ n = 18	-23.3±1.4‰ 1.0±.0.4‰ n = 11	-24.3±1.0‰ 1.7±.0.7‰ n = 4	**-23.6±1.3‰** **1.2±0.5‰** **n = 33**
**Barley** (*Hordeum vulgare)*	-22.9±0.9‰ N/A n = 4	-23.0±0.7‰ N/A n = 15	-23.2±0.7‰ N/A n = 8	-23.5±1.1‰ N/A n = 6	**-23.1±0.8‰** **N/A** **n = 33**
**Total**	**N = 24**	**N = 144**	**N = 135**	**N = 107**	**N = 410**

N/A = Not measured

Early Bronze Age fauna results are summarized in Table 2 and individually plotted in Fig 2. The dog δ^13^C (-19.2‰) and δ^15^N (7.4‰) values indicate consumption of a C_3_ terrestrial diet. Cattle have the most ^13^C-enriched results of the domestic animals (δ^13^C = -17.1±0.6‰), suggesting a component of C_4_ or marine plants in their diets. The unambiguously identified sheep (δ^13^C = -18.3±1.1‰; δ^15^N = 5.7±0.5‰) and goats (δ^13^C = -18.5±1.1‰; δ^15^N = 5.6±1.4‰) are isotopically indistinguishable, which is evidence of similar feeding regimes. While the mean δ^13^C results of the sheep and goats are more ^13^C-depleted compared to the cattle, some individuals also display a ^13^C-enrichment suggestive of a diet based on substantial amounts of C_4_ or marine plants. Thus, some sheep and goats were grazed and kept with the cattle. The tightly clustered δ^13^C (-19.2±0.3‰) and δ^15^N (6.9±0.6‰) results of the suids indicate they consumed a homogenous C_3_ terrestrial diet.

**Table 2:** Summary of the mean ± SD isotopic results of the humans and animals by time period from Tell Tweini. For the time periods and total columns, the first line = δ^13^C (mean ± SD); second line = δ^15^N (mean ± SD); third line = number of samples (n).

Human/Animal	Early Bronze Age	Middle Bronze Age	Late Bronze Age	Iron Age	Total
**All Humans**	-	-19.9±0.4‰ 6.7±0.7‰ n = (16) out of 44 (36.4%)	-	-	-19.9±0.4‰ 6.7±0.7‰ n = (16) out of 44 (36.4%)
**Dog** **(*Canis lupus* f. familiaris)**	-19.2‰ 7.4‰ n = (1) out of 1	-18.0‰ 8.6‰ n = (2) out of 2	-18.4±0.8‰ 6.5±0.8‰ n = (7) out of 7	-17.5±1.7‰ 7.2±1.1‰ n = (9) out of 10	**18.0±1.1‰** **7.1±1.1‰** **n = (19) out of 20**
**Cattle** **(*Bos primigenius* f. taurus)**	-17.1±0.6‰ 5.8±0.8‰ n = (5) out of 10	-18.9±1.2‰ 6.3±1.3‰ n = (4) out of 11	-18.8±1.6‰ 6.6±2.3‰ n = (9) out of 17	-17.9±1.6‰ 5.4±1.1‰ n = (11) out of 25	**-18.2±1.5‰** **5.9±1.6‰** **n = (29) out of 63**
**Suid** **(*Sus scrofa* f. domestica & *Sus scrofa*)**	-19.2±0.3‰ 6.9±0.6‰ n = (6) out of 10	-20.1‰ 6.5‰ n = (2) out of 3	-19.9±0.4‰ 5.2±0.9‰ n = (9) out of 10	- n = (0) out of 3	**-19.6±0.5‰** **5.9±1.1‰** **n = (17) out of 26**
**Sheep** **(*Ovis ammon* f. aries)**	-18.3±1.1‰ 5.7±0.5‰ n = (8) out of 11	-17.9±1.3‰ 6.1±1.9‰ n = (5) out of 10	-18.4±0.9‰ 6.1±1.5‰ n = (10) out of 12	-18.3±1.1‰ 5.8±1.3‰ n = (14) out of 23	**-18.3±1.1‰** **5.9±1.3‰** **n = (37) out of 56**
**Goat** **(*Capra aegagrus* f. hircus)**	-18.5±1.1‰ 5.6±1.4‰ n = (12) out of 12	-18.3±0.4‰ 4.9±1.0‰ n = (7) out of 9	-18.4±1.8‰ 5.5±0.8‰ n = (10) out of 13	-19.0±1.0‰ 5.9±1.6‰ n = (14) out of 22	**-18.6±1.2‰** **5.6±1.3‰** **n = (44) out of 56**
**Fallow deer** **(*Dama mesopotamica*)**	-19.6‰ 4.8‰ n = (1) out of 3	-20.4‰ 4.6‰ n = (2) out of 2	-19.3±0.9‰ 4.2±1.1‰ n = (8) out of 10	-19.6±0.4‰ 4.6±0.6‰ n = (8) out of 9	**-19.5±0.7‰** **4.4±0.9‰** **n = (19) out of 23**
**Brown hare** **(*Lepus europaeus*)**	-19.5‰ 4.8‰ n = (1) out of 1	-	-19.9±1.8‰ 4.6±2.4‰ n = (4) out of 4	-	**-19.8±1.5‰** **4.7±2.1‰** **n = (5) out of 5**
**Brown bear** **(*Ursus arctos*)**	-	-	n = (0) out of 1	-20.2‰ 7.5‰ n = (2) out of 2	**-20.2‰** **7.5‰** **n = (2) out of 3**
**Gazelle** **(*Gazella* sp.)**	-	-	-18.7‰ 5.1‰ n = (2) out of 2	-19.4±0.5‰ 5.5±1.0‰ n = (3) out of 3	**-19.1±0.7‰** **5.3±0.9‰** **n = (5) out of 5**
**Accipitrid bird of prey (Accipitridae)**	-	-	-19.0‰ 8.7‰ n = (1) out of 1	-14.3‰ 9.3‰ n = (1) out of 1	**-16.7‰** **9.0‰** **n = (2) out of 2**
**Black kite** **(*Milvus migrans*)**	-	-	-	-16.5‰ 8.2‰ n = (1) out of 1	**-16.5‰** **8.2‰** **n = (1) out of 1**
**Buzzard** **(*Buteo buteo*)**	-18.7‰ 10.8‰ n = (1) out of 1	-	-18.4‰ 8.2‰ n = (1) out of 1	-	**-18.5±0.2‰** **9.5±1.8‰** **n = (2) out of 2**
**Chukar partridge** **(*Alectoris chukar*)**	-18.5‰ 7.1‰ n = (1) out of 1	-	-19.6‰ 4.9‰ n = (2) out of 2	-19.1‰ 5.3‰ n = (2) out of 2	**-19.2±0.5‰** **5.5±1.1‰** **n = (5) out of 5**
**Crow** **(*Corvus* sp.)**	-18.4‰ 11.1‰ n = (1) out of 1	-18.8‰ 7.7‰ n = (1) out of 1	-19.2‰ 9.4‰ n = (1) out of 1	-18.6‰ 6.5‰ n = (1) out of 1	**-18.8±0.3‰** **8.7±2.0‰** **n = (4) out of 4**
**Duck** **(*Anas* sp.)**	-	-	-23.3±1.3‰ 7.7±0.9‰ n = (4) out of 5	-	**-23.4±1.3‰** **7.7±0.9‰** **n = (4) out of 5**
**Goose** **(*Anser* sp.)**	-18.7‰ 7.3‰ n = (1) out of 1	-	-19.8±0.4‰ 5.3±0.6‰ n = (3) out of 3	-20.0±0.5‰ 5.8±0.4‰ n = (8) out of 11	**-19.9±0.6‰** **5.8±0.7‰** **n = (12) out of 15**
**Heron** **(*Ardea* sp.)**	-	-	-18.6‰ 10.4‰ n = (1) out of 1	-	**-18.6‰** **10.4‰** **n = (1) out of 1**
**Pigeon** **(*Columba* sp.)**	-14.7‰ 6.1‰ n = (1) out of 1	-19.0‰ 4.5‰ n = (1) out of 1	-19.5‰ 7.5‰ n = (1) out of 1	- n = (0) out of 1	**-17.7±2.7‰** **6.1±1.5‰** **n = (3) out of 4**
**Total**	**N = 39 out of 53 (73.6%)**	**N = 25 out of 39 (64.1%)**	**N = 73 out of 90 (81.1%)**	**N = 75 out of 114 (65.8%)**	**N = 210 out of 296 (70.9%)**

The fallow deer (δ^13^C = -19.6‰; δ^15^N = 4.8‰) and brown hare (δ^13^C = -19.5‰; δ^15^N = 4.8‰) have the most ^13^C and ^15^N-depleted results and consumed C_3_ terrestrial foods. The buzzard (10.8‰) and crow (11.1‰) have the most elevated δ^15^N values (~3–5‰ higher than the domestic animals), which could suggest they fed on the carcasses of small animals or, in the case of the crow, even on human refuse at Tell Tweini. The pigeon has the most ^13^C-enriched value (-14.7‰) and this along with the low δ^15^N value (6.1‰) indicates that it consumed a mixed C_3_ and C_4_ diet. The goose results (δ^13^C = -18.7‰; δ^15^N = 7.3‰) are similar to the domestic animals (sheep, goats, dogs). However, it likely did not consume human waste as only wild species were identified at Tell Tweini: the white-fronted goose (*Anser albifrons*) and the grey-lag goose (*Anser anser*) [44] and geese domestication did not occur until the 2^nd^ millennium BC [60] or after the period of this specimen.

### Middle Bronze Age (ca. 2000–1600 BC)

**Fig 3:**
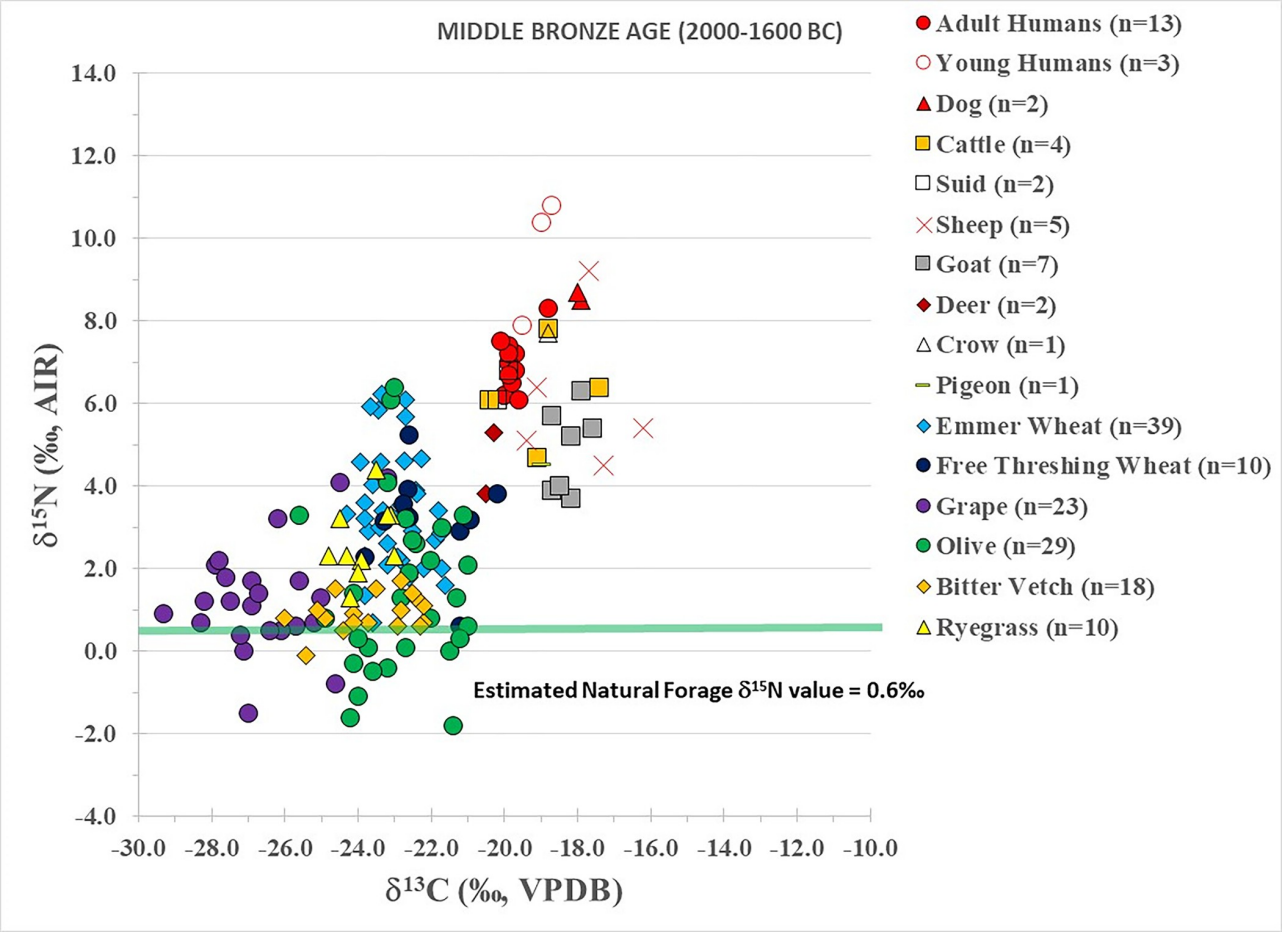
Middle Bronze Age plant, animal and human isotopic results from Tell Tweini. Estimated natural forage δ^15^N value calculated by subtracting 4‰ from the average δ^15^N values of the deer (e.g., [8]).

Middle Bronze Age plant results are summarized in Table 1 and individually plotted in Fig 3 (except for the barley). Plants have a wide range of mean δ^13^C values (-26.5±‰ to -22.0‰) but a narrow range of mean δ^15^N values (1.2‰ to 3.7‰). In particular, five plant species (emmer wheat, free threshing wheat, olive, rye grass, bitter vetch and barley) have mean δ^13^C results that cluster together between –23.8‰ to –22.0‰ whereas the grapes show a greater ^13^C-depletion (-26.5‰).

Middle Bronze Age fauna results are summarized in Table 2 and individually plotted in Fig 3. The two dogs have elevated δ^13^C (-18.0‰) and δ^15^N (8.6‰) results that plot above the adult humans and most of the domestic animals. Cattle (δ^13^C = -18.9±1.2‰; δ^15^N = 6.3±1.3‰), sheep (δ^13^C = -17.9±1.3‰; δ^15^N = 6.1±1.9‰) and goats (δ^13^C = -18.3±0.4‰; δ^15^N = 4.9±1.0‰) have similar δ^13^C and δ^15^N values, indicating these animals consumed predominately C_3_ based diets and were grazed together. However, like the Early Bronze Age, some cattle and sheep show an input of C_4_ or marine plants in their diets. Isotopic results of the two suids (δ^13^C = -20.1‰; δ^15^N = 6.5‰) are in the range of the domestic animals and nearly identical to the adult humans. Fallow deer have the lowest δ^13^C (-20.4‰) and δ^15^N (4.6‰) results and consumed C_3_ terrestrial diets. In contrast to the Early Bronze Age, the Middle Bronze Age pigeon (δ^13^C = -19.0‰; δ^15^N = 4.5‰) consumed a C_3_ diet. The crow (δ^13^C = -18.8‰; δ^15^N = 7.7‰) also had a C_3_ diet.

Human δ^13^C results (-19.8±0.3‰) indicate consumption of terrestrial C_3_ diets (Table 2 and Fig 3). However, the adult humans have low δ^15^N values (range = 6.1‰ to 8.3‰; 6.9±0.6‰) that are similar or slightly elevated compared to the domestic animals. The three children also consumed C_3_ terrestrial diets and two of these individuals had elevated δ^15^N values (>10‰) indicative of breastfeeding [61].

### Late Bronze Age (ca. 1600–1200 BC)

**Fig 4:**
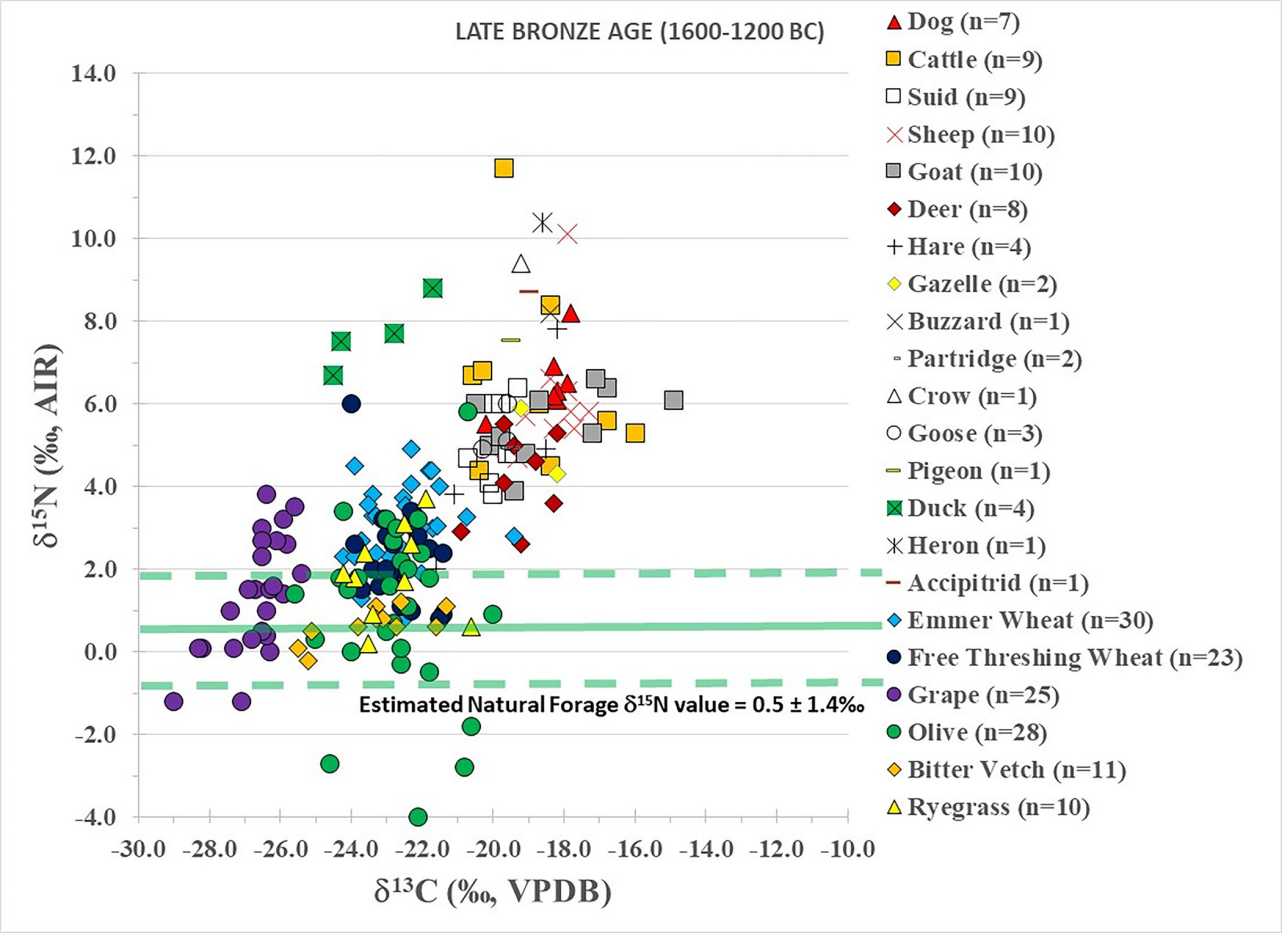
Late Bronze Age plant and human isotopic results from Tell Tweini. Estimated natural forage δ^15^N value calculated by subtracting 4‰ from the average δ^15^N values of the deer, hare and gazelle (e.g., [8]).

Late Bronze Age plants are summarized in Table 1 and individually plotted in Fig 4. Apart from the grapes (δ^13^C = -26.6‰±0.9‰; δ^15^N = 1.7±1.4‰), all of the Late Bronze Age mean δ^13^C plant results plot between -23.3‰ to -22.5‰ and the mean δ^15^N results plot between 1.0‰ to 3.3‰.

Late Bronze Age fauna results are summarized in Table 2 and plotted in Fig 4. Dog δ^13^C (-18.4±0.8‰) and δ^15^N (6.5±0.8‰) values plot with the other domestic animals and reflect a C_3_ terrestrial diet. The isotopic results of the cattle (δ^13^C = -18.8±1.6‰; δ^15^N = 6.6±2.3‰), sheep (δ^13^C = -18.4±0.9‰; δ^15^N = 6.1±1.5‰) and goats (δ^13^C = -18.4±1.8‰; δ^15^N = 5.5±0.8‰) are all tightly clustered. This indicates the domestic herbivores consumed similar diets and were likely kept together at Tell Tweini. However, as in the previous periods some cattle, sheep and goats have ^13^C-enriched values indicative of dietary contributions from C_4_ or marine plants. The suids have tightly clustered δ^13^C (-19.9±0.4‰) and δ^15^N (5.2±0.9‰) values again reflecting a homogenous C_3_ terrestrial diet as seen in the Middle Bronze Age. Fallow deer have δ^13^C (-19.3±0.9‰) values that cluster with the majority of the other terrestrial animals, but the δ^15^N (4.2±1.1‰) values are low and reflect the general wild vegetation consumed. The brown hare had a wide range of δ^13^C (-21.6‰ to -18.2‰; mean ± SD = -19.9±1.8‰) and δ^15^N values (2.0‰ to 7.8‰; mean ± SD = 4.6±2.4‰) indicating that they consumed C_3_ diets. Isotopic results of the gazelle (δ^13^C = -18.7‰; δ^15^N = 5.1‰) cluster with the other animals and indicate they consumed a C_3_ diet. Gazelles are herbivores that can have a varied diet but generally feed mostly on grasses [62].

Avifauna display variable isotopic results related to the specific habitats and diets of the species studied. The heron has the highest δ^15^N result (10.4‰) of all of the Late Bronze Age birds, and this coupled with its δ^13^C result (-18.6‰), indicates its diet was based on a predominately freshwater aquatic food chain. The crow (δ^13^C = -19.2‰; δ^15^N = 9.4‰), accipitrid bird of prey (δ^13^C = -19.0‰; δ^15^N = 8.7‰) and buzzard (δ^13^C = -18.4‰; δ^15^N = 8.2‰) all consumed C_3_ diets with high amounts of protein, including possibly carrion or human refuse. The ducks have the lowest δ^13^C values (-23.3±1.3‰) as well as elevated δ^15^N values (7.7±0.9‰), which reflect an aquatic freshwater habitat and diet. The isotopic results of the geese (δ^13^C = -19.8±0.4‰; δ^15^N = 5.3±0.6‰) and chukar partridge (δ^13^C = -19.6‰; δ^15^N = 4.9‰) are similar and show these species had C_3_ terrestrial diets. Finally, the pigeon (δ^13^C = -19.5‰; δ^15^N = 7.5‰) consumed a C_3_ terrestrial diet, as opposed to the Early Bronze Age, and is slightly ^15^N-enriched compared to the domestic animals and the suids.

### Iron Age (ca. 1200–333 BC)–predominantly Iron Age I/II (ca. 1200–700 BC)

**Fig 5:**
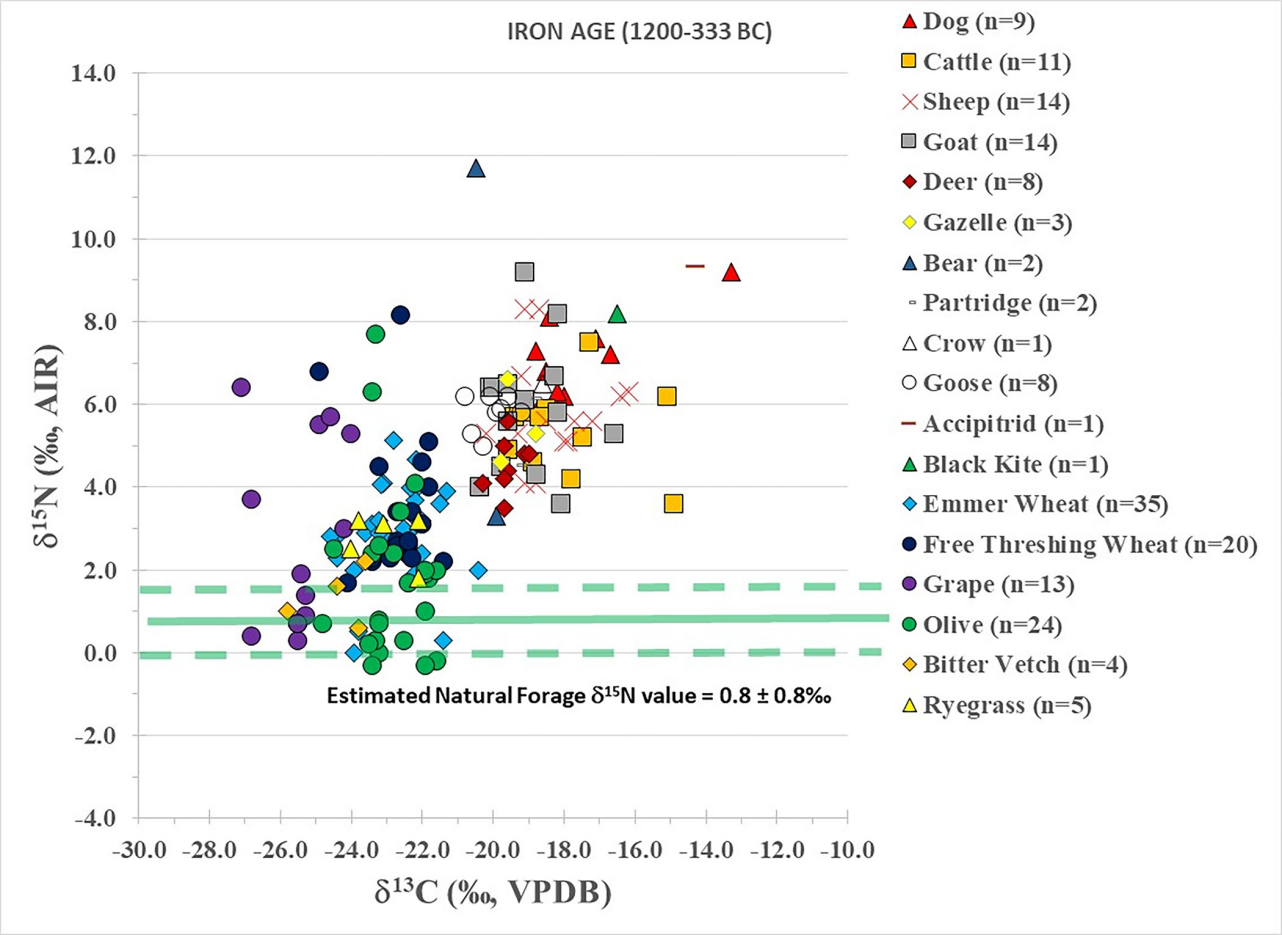
Iron Age plant and animal isotopic results from Tell Tweini. Estimated natural forage δ^15^N value calculated by subtracting 4‰ from the average δ^15^N values of the deer and gazelle (e.g., [8]).

Iron Age plants are summarized in Table 1 and individually plotted in Fig 5. Emmer wheat (δ^13^C = -22.6±0.9‰; δ^15^N = 3.3±1.2‰), free threshing wheat (δ^13^C = -22.6±0.8‰; δ^15^N = 3.8±1.7‰), and olives (δ^13^C = -22.7±0.9‰; δ^15^N = 2.1±2.0‰) have tightly clustered isotopic results similar to the wild ryegrass weed (δ^13^C = -22.9±0.9‰; δ^15^N = 3.1±0.6‰). Bitter vetch had isotopically depleted results (δ^13^C = -24.3±1.0‰; δ^15^N = 1.7±0.7‰) compared to the abovementioned plants. Grapes have the lowest δ^13^C (-25.4±1.0‰) but similar δ^15^N values (3.1±2.3‰) to all of the plants.

Iron Age fauna results are summarized in Table 2 and plotted in Fig 5. Dogs have δ^13^C (-17.5±1.7‰) and δ^15^N values (7.2±1.1‰) similar or only slightly elevated compared to the domestic animals as a whole. Interestingly, one dog has a δ^13^C value of –13.2‰ and a δ^15^N value of 9.2‰, which is evidence of significant consumption of C_4_ or marine protein. As this dog plots with many of the fish species found at Tell Tweini but not above these fish species [15], this possibly suggests a diet based on C_4_ resources. However, additional research, such as δ^13^C_AA_ analysis [63], is needed to confirm this. The cattle (δ^13^C = -17.9±1.6‰; δ^15^N = 5.4±1.1‰), sheep (δ^13^C = -18.3±1.1‰; δ^15^N = 5.8±1.3‰) and goats (δ^13^C = -19.0±1.0‰; δ^15^N = 5.9±1.6‰) show significant isotopic overlap indicating that at the population level they were fed or kept in a similar manner. However, like in the Bronze Age, some individual cattle (δ^13^C range = -20.6‰ to -14.9‰), sheep (δ^13^C range = -20.2‰ to -14.9‰) and goats (δ^13^C range = -20.4‰ to -16.6‰) consumed an input of C_4_ or marine protein in their diet. Fallow deer (δ^13^C = -19.6±0.4‰; δ^15^N = 4.6±0.6‰) and gazelle (δ^13^C = -19.4±0.5‰; δ^15^N = 5.5±1.0‰) have similar isotopic results and both fed on C_3_ terrestrial resources. The two brown bears have similar δ^13^C results (-19.9‰ and -20.5‰) but different δ^15^N values (3.3‰ and 11.7‰). Inspection of the skeletal remains found that the individual with the low δ^15^N result was a young adult with permanent teeth that were unworn, whereas the individual with the high δ^15^N result was an adult. Thus, these differences in protein consumption between these two bears (herbivore vs. high trophic level carnivore), possibly reflect different diets during the life stages of this species (e.g., [64]).

Iron Age birds show a wide range of isotopic results (Fig 5). The accipitrid bird of prey (δ^13^C = -14.3‰; δ^15^N = 9.3‰) and the black kite (δ^13^C = -16.5‰; δ^15^N = 8.2‰) are elevated in terms of δ^13^C and δ^15^N results. This indicates these birds fed on mixed C_3_/C_4_ diets (possibly derived from preying on wild small terrestrial vertebrates or domestic animals in the form of human refuse or carrion) or mixed C_3_/marine protein diets. The chukar partridge (δ^13^C = -19.1‰; δ^15^N = 5.3‰), crow (δ^13^C = -18.6‰; δ^15^N = 6.5‰) and geese (δ^13^C = -20.0±0.5‰; δ^15^N = 5.8±0.4‰) all show relatively similar δ^13^C and δ^15^N values and consumed C_3_ diets.

## Discussion

### Archaeobotanical assemblage

Modern natural vegetation in the area belongs to the Mediterranean phytogeographical zone [65], which today is mostly degraded to cultivated areas, pasture lands or maquis shrublands. The archaeobotanical assemblage is dominated by annual crops (cereals and pulses) and their weeds (e.g., ryegrass), as well as fruits like grapes, figs and olives [31] (S1 Fig in S1 File). Beside cultivated plants, a variety of wild plants were recovered. Looking at the ecological information derived from the archaeobotanical assemblages the highest proportion of open steppe vegetation, ruderal habitats and marshlands species occurs during the Iron Age (S2 Fig in S1 File), albeit they are found throughout all occupation periods. The archaeobotanical indicators for these habitats likely originate from the dung fuel used at Tell Tweini and reflect the seeds/fruits of the surrounding vegetation grazed by the domestic animals. The higher input of such plants in the Iron Age could be related to more intensive use of the surroundings, and the input of C_4_ plants from disturbed habitats (*Digitaria*, *Echinochloa*, *Heliotropium*, *Portulaca*, *Setaria*) and wetlands (*Aeluropus*, *Eleocharis*). The archaeobotanical assemblages preserved in the charred state yielded only a small part of the plants that are potentially indicative for such habitats. Some of the C_4_ taxa potentially growing near Tweini have tiny seeds with nearly no chance to survive charring and adverse taphonomic conditions. Furthermore, some C_4_ plants may not have been recognized due to preservation and identification limitations, like some species of the grass (Poaceae) or sedge (Cyperaceae) families. Finally, indirect evidence for the presence of habitats suitable for C_4_ seed plants is the high diversity of ruderals [44]–i.e., indicators for areas exposed to anthropo-zoogenic impact.

### Diachronic plant isotopic patterns

Since plant species differ in their physiology, their isotopic ranges also diverge [66]. For example, carbon fixation in annual species, such as cereals, takes place within a very short period of the final growth cycle and thus reflects limited seasonal conditions mostly from May to July. In contrast, tree species preserve an extended climate signal in their fruits. Ecological requirements of the different species result in different responses to reduced water availability, e.g., emmer wheat being more drought resistant than free threshing wheat [67].

### Emmer & free threshing wheat

Emmer wheat was the main cereal crop at Tell Tweini during the Middle and Late Bronze Ages, while free threshing wheat is nearly absent and only becomes more important during the Iron Age (S1 Fig in S1 File). Emmer wheat is relatively drought resistant compared to free-threshing wheat, but it starts to disappear in the Near Eastern inland sites from the Early Bronze Age on, although it remains a major crop in most of the Mediterranean until including the Late Bronze Age. For the Middle Bronze Age Levant, emmer wheat appears to be more ubiquitous in the south [67]. The less drought resistant free threshing wheat, which may have received irrigation in Mesopotamian sites, occurs in much smaller proportions and lower ubiquities in the Middle Bronze Age layers than in the later periods of Tell Tweini. Its ubiquities increase in the Late Bronze and Iron Age, especially in the Iron Age-II period, which corresponds with the general Near Eastern pattern and might be related with changes in crop husbandry techniques and/or the supra-regional economic development of markets in the Near East and the Mediterranean during the Late Bronze Age and the Iron Age [68, 69].

**Fig 6:**
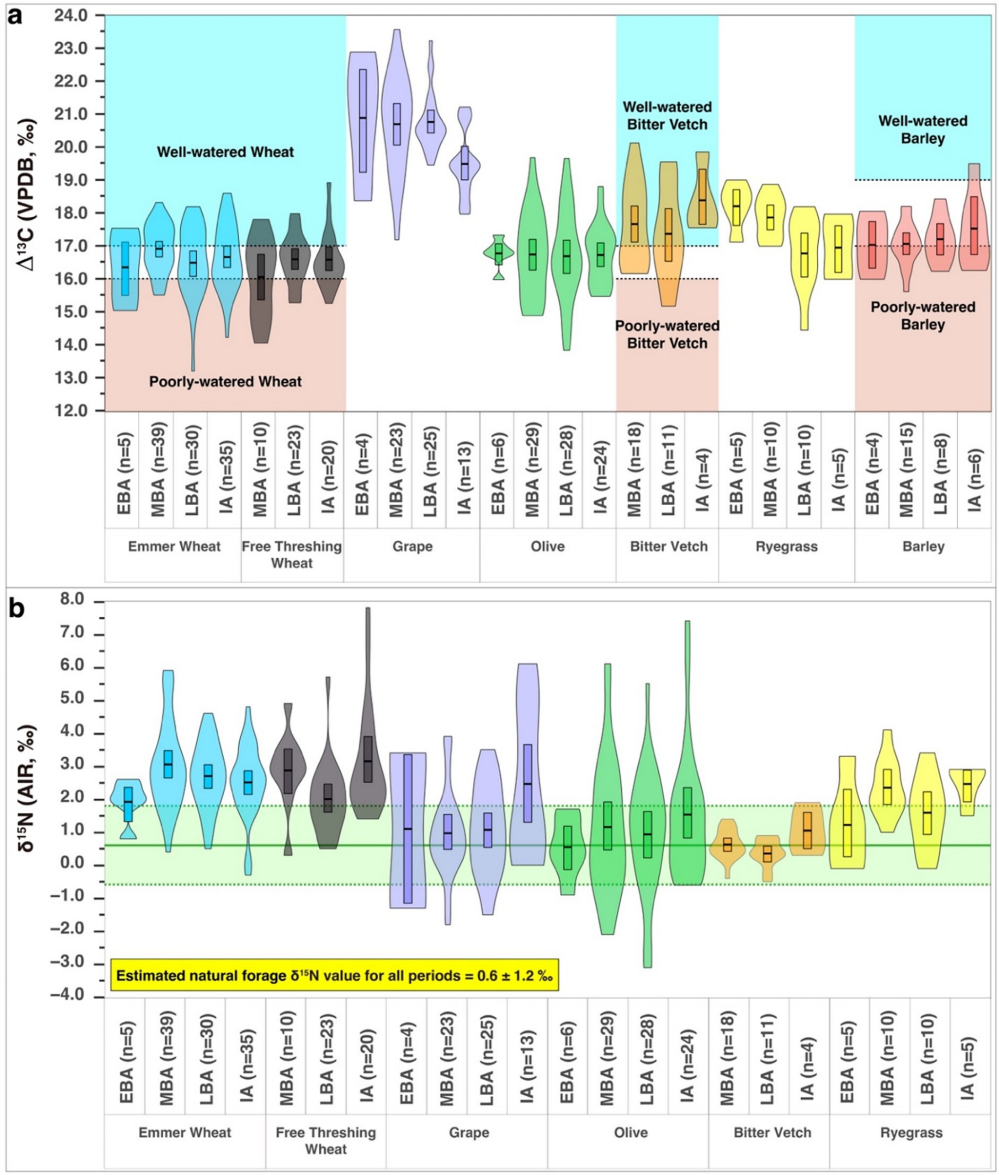
Diachronic plant isotopic results from Tell Tweini. (a) Violin plots of the diachronic Δ^13^C values calibrated with the AIRCO2_LOESS program [55, 56] for plant species at Tell Tweini. Horizontal dashed lines reflect approximate water status Δ^13^C boundaries determined by Wallace et al. [85, 86]. (b) Violin plots of the diachronic δ^15^N values for plant species at Tell Tweini. Estimated natural forage δ^15^N value (mean ± SD) calculated by subtracting 4‰ from the average δ^15^N values of the wild animals (deer, hare and gazelle; [8]). Emmer and free-threshing wheat results are elevated during all periods of study and reflect that manure was used as a fertilizer for these crops. Boxes in the middle of the violins represent the mean δ^13^C and its 95% confidence intervals for each animal group (Plots generated using RStudio V.1.4.1717 with final layout created using Adobe Illustrator CC 2019 V.23.1.1).

According to the Δ^13^C results, the emmer wheat received adequate amounts of water during all periods at Tell Tweini (Fig 6A). In particular, the best water status for the emmer wheat was during the Middle Bronze Age, which compares well to results from the Middle Bronze Age site of Qatna, Syria roughly 140 km southeast of Tweini [70]. The Δ^13^C values remain relatively stable during the Late Bronze and Iron Age, although slightly decreased compared to the Middle Bronze Age values and are similar to those of the Early Bronze Age as well. It should be noted that we, generally summarized all isotopic measurements under broad chronological categories due to the lack of high-precision dating and uneven distribution of samples in the diverse settlement phases, e.g., many of the archaeobotanical samples are assigned as Iron Age I/II, rather than to Iron Age I or Iron Age II. Furthermore, some crop taxa assemblages only contain either Iron Age I or Iron Age II samples, which limits a comparative approach. However, if we consider Iron Age I, which has been attributed to the time range of the 3.2 kyrs BP event, separately from Iron Age II, we can see differences in the Δ^13^C values for emmer wheat, where the means for Iron Age I are 16.4±1.2‰ and those for Iron Age II are 17.2±0.9‰. However, these differences are not statistically significant (Independent-samples t-test, p = 0.211). For free-threshing wheat (Iron Age I– Δ^13^C = 16.8±1.2‰; Iron Age II– Δ^13^C = 16.7±1.0‰), no such difference exists and considering the only moderate, statistically insignificant stress result of 16.4‰ for Iron Age I emmer and the lack of drought stress in free-threshing wheat, the Iron Age farmers appear to have successfully applied cultivation strategies advantageous for these crops.

The Early Bronze Age data from Tell Tweini contrast to some of the other Levantine sites, such as Tel Kabri, where the mean Δ^13^C of the emmer is 15‰ [71, 72], which might reflect differing socio-economic attitudes of different societies. For all periods, an overwhelming portion of the emmer wheat received adequate amounts of water (84 out of 109; 77%) and just under half were well watered (47 out of 109; 43%) (S3 Fig in S1 File). In terms of δ^15^N, emmer wheat display some of the most elevated values for all periods of study, similar to the free threshing wheat (Fig 6B). All mean δ^15^N results are elevated compared to the estimated natural forage, which suggests that some form of manuring occurred for this crop during all periods at Tell Tweini (e.g., [8]). This seems quite reasonable as stubble fields of wheat are welcome browsing ground for small ruminants. However, looking at the individual data points, the majority are strikingly elevated (S3 Fig in S1 File), and suggest the likelihood that the addition of manure as a fertilizer was more deliberate and significant in terms of how the emmer wheat was managed.

Considering all time periods, 75% (40 out of 53) of the free-threshing wheat specimens studied here received adequate amounts of water and among these 26% (13 out of 53) were well watered (S4 Fig in S1 File). Free-threshing wheat appears to diachronically reflect the opposite trend compared to emmer wheat, i.e., the Middle Bronze Age mean Δ^13^C value bordering on poorly watered status but showing improved conditions during the Late Bronze and Iron Ages (Fig 6A), which is in contrast to the findings at Qatna, Syria, further south [70]. Water-improved growing conditions for free-threshing wheat at Tweini can be interpreted in three different ways. They may (1) represent a climate pattern of increased aridity during the earlier part of the Middle Bronze Age I that improved during the Late Bronze Age, (2) the opposite climate trend, i.e., continuously increasing aridity, which was counteracted by irrigation or placement of fields close to or within the extent of the wetland area or (3) economically motivated irrigation for improving yields. The same long-term pattern is visible in barley, but not in emmer. However, emmer was losing its economic importance in the ancient Near East throughout time, and this might be reflected in the slightly decreasing Δ^13^C means after the Middle Bronze Age and also in its decreasing ubiquity, possibly related to less care of its moisture availability, which furthermore, may support a slight climatic drying trend (Fig 6A).

The data trends of the wheat species may be best explained by shifting preferences of production and consumption of emmer wheat with the end of the Middle Bronze Age to free-threshing wheat in the Iron Age, which is also supported by the changing archaeobotanical ubiquities of these two taxa (S1 Fig in S1 File), and by the general development of emmer cultivation in the Near East [67, 68]. Comparing the trends of free-threshing wheat with those of barley (Fig 6A), which has Δ^13^C means near the poorly watered threshold in the Early and Middle Bronze Age but developing into more moderate conditions in the Late Bronze and Iron Age, it appears likely that moisture availability improved during these two periods, either through generally moister local conditions or through increased care for sufficient soil moisture.

Similar to the emmer wheat, the free-threshing wheat δ^15^N means are elevated above the estimated natural forage for all time periods (Fig 6B). This is generally considered evidence animal manure was used as a fertilizer for wheat (S4 Fig in S1 File) [8]. Taking into account that foliar δ^15^N increases with decreasing mean annual precipitation [73, 74], Styring et al. [75] proposed a model for the relationship between cereal grain δ^15^N values from different manuring levels and mean annual precipitation ranges. Since past mean annual precipitation is unknown, using modern values may help us better understand what δ^15^N range may be expected to indicate manuring. Mean annual precipitation at Jableh is approximately 800 mm and applying this value to the model by Styring et al. [75] produces δ^15^N ranges for low level manuring of ca. 1–2‰, 2–7‰ for medium level manuring and above 7‰ of high manure input. For the crop values from Tell Tweini, this implies that high manure input was never practiced, whereas medium level manuring of cereals likely took place. This pattern is visible in the relatively high values in both Middle Bronze Age wheat species, pointing to medium level manure input (Fig 6B). An alternative interpretation may be the shifting to new agricultural lands with higher nitrogen levels.

The Iron Age free-threshing wheat δ^15^N results (3.1±1.7‰) are even more elevated than those of the Late Bronze Age (2.0±1.1‰), and this difference is statistically significant (Mann-Whitney U Test, p = 0.009). This may either point to increased manuring, cultivation on more fertile soils than before or a combination of decreased moisture availability and the other factors mentioned. Since marshland plant species are particularly abundant in the Iron Age samples (S2 Fig in S1 File), it seems highly plausible that an extension of the land use area took place during the Iron Age. Keeping the increase of δ^15^N with decreasing mean annual precipitation in mind, the extended duration of Cold Period 3 (acc. to [29]) alongside the long-term Holocene increase in aridity might be reflected in the generally higher δ^15^N values of Iron Age crop species, including free-threshing wheat, grape, olive and bitter vetch, respectively.

### Grapes

While plenty of comparative studies for cereals exist, there is virtually no comparative isotopic data available for ancient vine and tree crops such as grapes and olives. Therefore, we can currently only compare the Tell Tweini data to unpublished data of Simone Riehl from other Near Eastern sites (S5a Fig in S1 File).

At Tell Tweini, grapes have the highest Δ^13^C results for all periods (Fig 6A). Studies on the natural abundance of stable isotopes in leaves, berries and pulp of modern grape cultivars indicate δ^13^C values below –26‰, which equal Δ^13^C values >17‰, reflect an absence of water deficits, whereas δ^13^C values higher than –26‰ (⁓Δ^13^C less than <16‰) occur under moderate to severe water deficit conditions [76, 77]. However, ancient data of unpublished Δ13C values indicates that the so far lowest values are evident from the Middle Bronze Age grapes of Tell Mozan in northern Syria (mean value of 17.9‰, Riehl unpublished), which, if comparable to modern values of vegetative materials, would indicate relatively good moisture conditions even for these lowest ancient seed materials (S5a Fig in S1 File). All grape values from Tell Tweini plot far above this level, even the comparatively low values for the Iron Age (mean value of 19.5‰) (S5a Fig in S1 File). The seeds for these measurements exclusively derive from Iron Age I samples, i.e., from a period that is considered to fall into a highly arid phase, and thus might indeed represent a considerable water stress for the grape trees at that time. However, comparing the Δ^13^C record for grapes at Tell Tweini with other Near Eastern archaeological sites throughout the Bronze and Iron Ages, the data suggest particular care for the cultivation of this crop species, since none of the Tell Tweini samples indicate water deficits, although the overall trend at Tell Tweini develops into lower water availability in the Iron Age (S5a Fig in S1 File; 6a Fig). Thus, Tell Tweini nicely integrates with other sites that share similar climate regimes (e.g., Tell Kabri in coastal northern Israel [72], Tell Fadous-Kfarabida on the Lebanese coast, Kinet Höyük and Tell Atchana in the Hatay Province in southern Turkey [12]) (S5a Fig in S1 File).

The diachronic Δ^13^C pattern of the ancient grapes at Tell Tweini indicates relatively stable water availability during the Bronze Age, with possible drier conditions, but no obvious water deficits in the Iron Age (Fig 6A and S5a Fig in S1 File). It is likely the inhabitants of Tell Tweini may have generally suffered less from aridification due to the abundant springs and rivers nearby. Based on the diachronic δ^15^N pattern there is little evidence the vineyards were systematically manured during the Bronze Age (Fig 6B). In the Iron Age the grapes show an increase in δ^15^N, and while this is not statistically significant (p > 0.06), the mean is above the natural forage which could indicate some addition of manure or be related to increased aridity, since at least the foliar δ^15^N increases with decreasing mean annual precipitation (see discussion of the cereals). As the increase in δ^15^N corresponds to a significant decrease in Iron Age I Δ^13^C values (Independent-samples t-test; p = 0.0), the evidence suggests the grapes are reflecting more a climatic and less manuring isotopic signal at Tell Tweini during the Iron Age I. Evidence in support of the possibility of water deficits affecting grape cultivation in relation with drier climatic conditions comes from the palynological studies in the region [27, 31]. In particular, a pollen core from the alluvial deposits of the Rumailah River found a large-scale shift to more arid conditions during the Iron Age [28]. However, it must be stressed that given the lack of comparative data for archaeological and in particular for modern grapes, it is difficult to disentangle whether this isotopic signal is fully reflecting aridity, a change to more intensive manuring or both [78]. What is clear is that despite a continuous slight decrease the ubiquity, and thus importance of the grapes, remains generally high in the Late Bronze Age and Early Iron Age (S1 Fig in S1 File). This fits the general patterns of fruit tree cultivation in the Levant, and indicates the cultural connotation and commercial range of grapes during these times [79].

### Olives

Olives are comparatively high in ubiquity during all periods at Tell Tweini and the neighboring Tell Sukas [80]. Ubiquities are however decreased during the Late Bronze Age and reach their highest values during the Iron Age, supporting the archaeologically based assumption of a considerable importance of olive processing and/or oil production during this time [20] (S1 Fig in S1 File). They have steady mean Δ^13^C values during all periods, as well as similar Δ^13^C values to the annual crop species, suggesting the olives did not appear to suffer drought stress throughout the whole Early Bronze to Iron Age sequence (Fig 6A), which is in line with their general ecophysiology. A diachronic comparison of olive Δ^13^C values from Tell Tweini indicates comparable data ranges with other Near Eastern sites (S5b Fig in S1 File), whereas particularly the Early Bronze Age values at other settlements further south are much lower, indicating that water availability for olives at Tell Tweini was exceptionally good during this period. This is supported by Ehrlich et al. [81] who analyzed Δ^13^C values in more than 500 olive pips from 51 archaeological settlements in Israel in geographic relation to reconstructed mean annual precipitation, based on the Soreq speleothems. They could elaborate a threshold Δ^13^C for olive trees under severe drought stress below 15.5 ± 0.5‰ which, applied to the olive pips from Tell Tweini, would support the generally favorable moisture conditions at the site (S6 Fig in S1 File). However, the data for modern olives [81] also show that values above 16.5‰ are usually reached only under irrigation within the region considered. Nevertheless, these values cannot be directly transferred to other regions since they require joint consideration with the regional aridity index. Olive δ^15^N values at Tell Tweini are more variable compared to the Δ^13^C values and are most elevated during the Iron Age (Fig 6B), suggesting either the previously assumed manuring of soils, indirectly through increased herding of the small ruminants or directly through dung input over extended regions of agricultural land, or may indicate the origin of olives from extended plantations during the Iron Age.

Excavations at the site indicate that the production of olive oil became a main economic activity of Tell Tweini and installations related to this activity could be found in every house during the Iron Age (see [82] 47, fig. 54). This is also the period, when generally, proportions and finds of olives in the Near East are higher, suggesting an ongoing intensification of olive production during the Iron Age [67]. This is in contrast to the study of a pollen-profile in the vicinity of the site, which interpreted the *Olea*-pollen-abundances to represent pollen from uncultivated trees [83]. Olive pollen percentages are high starting roughly before 2000 BC until the end of the Late Bronze Age (ca. 1200 BC), where there is a short low percentage, in line with climatic aridity and the fall of the Ugarit Kingdom, and is then followed by another high peak between roughly 1100–900 BC, which might be correlated with the flash precipitation reconstructed for later Iron Age I [28]. However, after this period olive pollen percentages go down again, stay low throughout most of the Iron Age II and are followed by a higher peak only in the later Iron Age III, the Persian Achaemenid Period. Even though our Δ^13^C data does not have a proper chronological resolution into the three different Iron Age phases, i.e., apart from Iron Age I and III samples, we only have some from Iron Age I/II, there is no difference in the Δ^13^C values between these phases. Thus, we are not able to resolve the question of whether the Iron Age II inhabitants of Tell Tweini processed olive fruits from wild or cultivated trees. Given the very high percentages and ubiquities of olive stones, as well as the generally good water availability for the trees, the presence of installations for olive processing in almost every household of the Iron Age II supports that the Tweini settlers were indeed heavily using these fruits during a period when the pollen percentages were low. Whether this means that the olives came from cultivated trees outside the pollen catchment area or whether we might expect some limitations of the pollen record (for example change in sedimentation rates biasing the pollen-abundance values) cannot be answered through this study.

In sum, the great diachronically stable economic importance of olives to Tell Tweini is not least owed to its ecophysiological stability and is likely the reason why no isotopic evidence of water stress is found. Furthermore, water sources would have been allocated near the olive groves, thus guaranteeing continuous water availability in times of drought. Regardless, it is clear that additional isotopic research needs to be focused on the crop management practices of olives in the Near East given their economic, cultural and dietary significance.

### Bitter vetch

Bitter vetch is a relatively drought tolerant pulse, frequently found in Near Eastern and Mediterranean archaeological sites. Starting with a ubiquity of 67% in the Middle Bronze Age at Tell Tweini, it decreases during the following periods (S1 Fig in S1 File). It was very well-watered during all periods (Fig 6A; 31 out of 33; 94%). The good water status of this crop at Tell Tweini, likely illustrates its importance as a dietary staple for the population, which was reflected in its frequent inclusion as a burial offering for the dead [84]. Comparison of the Tell Tweini Δ^13^C results to other sites in the Eastern Mediterranean shows that pulses were generally well cared for in terms of water availability [85, 86]. In particular, Tell Tweini has similar bitter vetch results to those of Assiros Toumba in Greece and Tell Nebi Mend in western Syria but elevated Δ^13^C results compared to Khirbet Fâris [85]. Furthermore, the observed patterns can be the result of intercropping, a common practice in traditional agricultural systems. In particular, the intercropping of crops that do not fix nitrogen, for example cereals with legumes is regarded as yield-increasing. Considering the watering status of olives (see the previous section) the intercropping of annual and perennial crop species appears plausible at Tell Tweini.

During the Iron Age bitter vetch has the highest Δ^13^C and δ^15^N values. While the number of specimens studied was small, this suggests that cultivation conditions improved for the crop, in terms of water and nitrogen availability. Since pulses are nitrogen-fixers, they are less affected by soil ^15^N-enrichment factors than cereals and have atmospheric values, i.e., around 0‰ [87, 88]. Based on this, Vaiglova et al. [89] concluded that if δ^15^N values of pulses are increased, they must have been manured under a high‐intensity regime. At Tell Tweini, the mean δ^15^N values range around 1‰ (Fig 6B), but it has to be taken into account that in arid regions, where nitrogen systems are more open, nitrogen fixation in the soil is inhibited by soil dryness and high temperatures [90], which leads to increased δ^15^N values in the plant matter [75, 91]. It is therefore difficult to conclude whether the δ^15^N values for bitter vetch at Tweini are slightly increased by possible cultivation on comparatively dry plots or through manuring. Since they, however, are not indicating drought stress, it is likely that dung input may have played a certain role during the Iron Age.

### Wild ryegrass

Wild ryegrass (*Lolium* sp.) Δ^13^C results decrease from the Early and Middle Bronze Age to the Late Bronze and Iron Age (Fig 6A). The Middle to Late Bronze Age difference is statistically significant (Independent-samples t-test, p = 0.015). As ryegrass is a weed that can grow with different crop species, including such that are not included into our stable isotope analysis, the change in Δ^13^C values from the Middle to the Late Bronze Age likely indicate that ryegrass grew with a crop species under reduced moisture availability. Its Δ^13^C values are similar to those of the bitter vetch in the Middle Bronze Age, whereas they resemble those of the emmer and free-threshing wheat during the Late Bronze and Iron Age. For the diachronic δ^15^N trend, similarities exist between ryegrass and free-threshing wheat in the Middle Bronze, Late Bronze and Iron Age samples (Fig 6B), suggesting that the weed was particularly associated with the free-threshing wheat. This is partially supported by the archaeobotanical records where the finds of *Lolium* are mainly associated with the threshing remains of wheat (emmer and to less extend free threshing wheat) and barley. Sufficient moisture availability for ryegrass is also supported by its occurrence with *Phalaris* (cf. *minor*) and *Polypogon* sp. in the archaeobotanical assemblage, both usually associated with wet and fresh growing conditions.

### Barley

Barley is a drought resistant crop that is abundant and ubiquitous in the Near East during the periods of study, and ancient texts from this region generate a model of Mesopotamian societies that are basically barley-producing societies, i.e., cultivating barley monocultures [67, 92]. Its considerable drought tolerance plausibly excludes it from large-scale irrigation, which is also supported by ancient texts [93]. At Tell Tweini, it was a main and ubiquitous cereal crop during all periods of study (S1 Fig in S1 File), but mostly during the Middle Bronze Age, whereas decreasing in importance in the early Iron Age, when free-threshing wheat becomes most abundant [44]. The Δ^13^C values of barley slightly increase throughout time, with all taking a position of moderate to no water stress. As discussed above the general trend corroborates that of the wheat species. Severe water stress is not indicated in any of the periods, which likely is owed to the position of the site near the Mediterranean coast and the vicinity of ground water sources. The supra-regional comparison with Δ^13^C values from other sites in coastal regions (e.g., Tell Fadous-Kfarabida in Lebanon, Tell Atchana and Kinet Höyük in Turkey) indicates close similarities and contrasts with values from sites further inland, where moisture availability was more limited [12].

### Archaeozoological assemblage

Fauna composition at Tell Tweini is in general agreement with what is found at contemporary sites in the northern Levant [44]. There was a limited use of (local) wild animal resources and the faunal remains were largely dominated by domesticated animals, mainly sheep, goats and cattle. Diachronic changes in the relative proportion of the major domestic animal species at Tell Tweini are minimal (S7 Fig in S1 File). Additional domestic species present at the site include: dog, donkey (*Equus africanus* f. asinus), horse (*Equus ferus* f. caballus) and dromedary (*Camelus thomasi* f. dromedarius). Wild game represents only a few percent of the identified macromammals at Tell Tweini [44]. Their importance seems to fluctuate somewhat through time, with the highest proportions occurring during the Late Bronze Age [44]. Marom and Bar-Oz [94] argue that in the Bronze and Iron Age of the Levant, hunting was a device of politics and power rather than subsistence; more game can be found in urban than rural contexts. Wild mammals identified at Tell Tweini include species that were presumably consumed, like hare, brown bear, wild boar, goitred gazelle, red deer (*Cervus elaphus*) and Mesopotamian fallow deer. Red fox (*Vulpes vulpes*) was also identified, but such taxon is more typically hunted for its fur (cf. [95]). Elephant (*Elephas maximus/Loxodonta africana*) is represented by a molar, perhaps collected as a rarity and hippo (*Hippopotamus amphibius*) by pieces of ivory that represent waste of artisanal activities. These species did not necessarily occur in the Tell Tweini region as their remains may have been brought in from elsewhere as raw material or a curiosity. Examination of previous elephant finds from Syria suggests that the Orontes Basin, including the Ghab Basin that is at about 30 km east of Tell Tweini, may have been an ideal environment for elephants [96]. Hippos may also have lived in the Orontes Basin and likely also occurred on the coastal plains of the southern Levant [97].

The recovered species indicate that animals were brought into the settlement from diverse habitats, including steppe environments (gazelle), but probably mostly woodland (fallow deer, wild boar). According to Kaniewski et al. [31], the closest forest to Tell Tweini were only a few kilometers away and were composed of oak woodland and warm mixed forests which show a decreasing trend in the Iron Age. In the faunal assemblages from Tell Tweini gazelles appear only in the Late Bronze Age and increase in relative importance in the subsequent Iron Age. As find numbers of wild mammals are so low it is unclear if this is an aleatory fluctuation or if there is a link with the arid event in the earlier Iron Age. Remains of birds are rare compared to those of mammals, but several taxa were identified including: ducks and geese, which are the most common group and chukar partridge and pigeon. While the latter bird taxa were presumably consumed, the others like the accipitrid birds of prey, black kite and buzzard may rather be carcasses (cf. [95]), although no complete or partially articulating skeletons were found. The predominance of water birds, suggests that some fowling was done in the reed marsh near Tell Tweini.

### Diachronic domestic animal isotopic patterns

**Fig 7:**
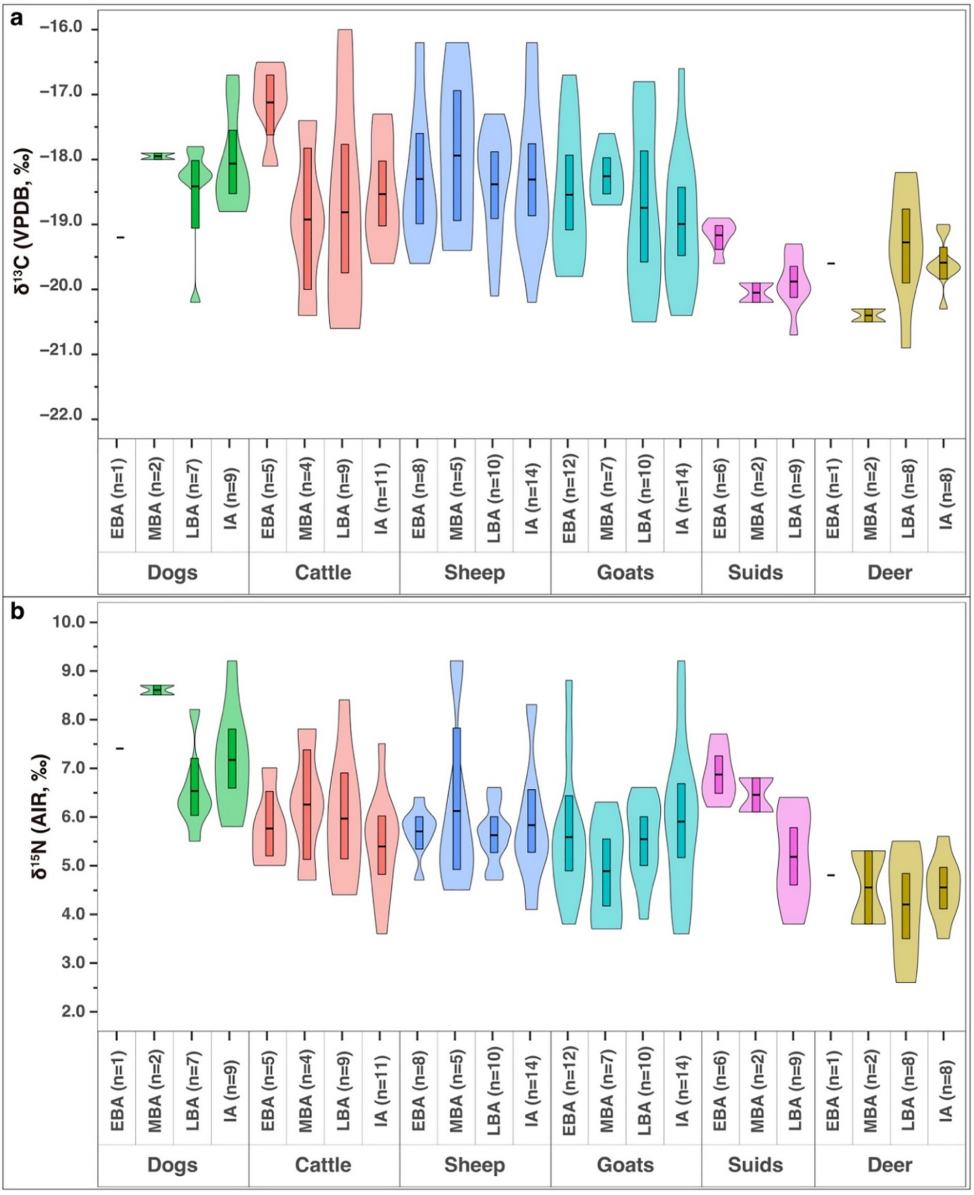
Diachronic animal isotopic results from Tell Tweini. Violin plots of the diachronic (a) δ^13^C and (b) δ^15^N results of the major animals at Tell Tweini. Boxes in the middle of the violins represent the mean results with 95% confidence intervals (Plots generated using RStudio V.1.4.1717 with final layout created using Adobe Illustrator CC 2019 V.23.1.1).

The dogs, cattle, sheep and goats produced sufficient numbers of isotopic results from each of the four time periods to investigate diachronic trends in the data (Fig 7A and 7B).

### Dogs

Dog remains from Tell Tweini were not numerous and with one exception do not show butchery marks. Thus, it is assumed that the dogs were not eaten by humans. Only a small number of Early (n = 1) and Middle Bronze Age (n = 2) dogs were measured so it is impossible to accurately compare these periods to later periods (Fig 7A and 7B. From the Late Bronze to Iron Age, the dogs have mean δ^13^C values that slightly decrease (~0.9‰), but this difference was not statistically significant (Mann-Whitney U Test; p = 0.79). The few Early and Middle Bronze Age dogs have elevated δ^15^N results above the domestic animals (Fig 7B). Thus, these dogs mainly consumed domestic animals during these periods, likely in the form of refuse from human activity [98]. During the Late Bronze Age, the dog δ^15^N results are similar to the domestic animals reflecting a reduced consumption of domestic animals and a possible increase in wild game in the diet such as deer. From the Late Bronze Age to the Iron Age, the dog δ^15^N results show an increase of 0.7‰, but this is not statistically significant (Independent-samples t-test; p = 0.214).

### Cattle

Cattle mean δ^13^C results and ranges during the four time periods show considerable variation (Fig 7A). Early Bronze Age cattle are elevated by 1.8‰ compared to the Middle Bronze Age, and this difference is statistically significant (Independent-samples t-test; p = 0.023). This indicates the cattle were fed or allowed to graze in areas with considerable amounts of C_4_ or possibly marine plants. Interestingly, no millet was recovered at Tell Tweini, so it is unlikely this was the source of the C_4_ component of the diet. However, the site was surrounded by wetland and coastal marshes with salt tolerant C_4_ grasses and shrubs, and animals grazing near the coast on these plants are expected to have ^13^C-enriched results (e.g., [99, 100]). Cattle with similar δ^13^C results reflecting C_4_ plant consumption were also found at the Early Bronze Age site of Archontiko, Greece [8]. As with Tell Tweini, no millet was found at Archontiko so it was argued these ^13^C-enriched results were the result of grazing on coastal salt marshes away from the site. During the Middle and Late Bronze Age, the δ^13^C results are lower, signifying a shift to a diet higher in C_3_ forage and possibly indicating a change in animal husbandry during these two periods where the cattle were kept closer to the site. Iron Age cattle again show a δ^13^C increase of ~1‰, but given the large standard deviation, these results are indistinguishable from the Middle and Late Bronze Ages and not statistically significant (Independent-samples t-test; p = 0.221). In contrast, the δ^15^N results of the cattle are relatively constant (range 5.4‰ to 6.6‰) over the four time periods, indicating that there was little change in the protein consumption or trophic level of these animals through time at Tell Tweini (Fig 7B).

### Sheep & goats

Sheep and goats are the most common animals recovered and are almost twice as abundant as cattle bones throughout all the periods at Tell Tweini (S7 Fig in S1 File) [44]. Presumably, in the surroundings of a large settlement such as Tell Tweini, it must have been easier to keep sheep and goat than cattle, since cattle are ecologically demanding animals which require good pasture and sufficient drinking water. Diachronic size variations were noted for the sheep and goats, but none were statistically significant [44]. In terms of δ^13^C, sheep and goats are relatively similar to each other during the four periods of study (Fig 7A). Thus, the sheep and goats had similar diets and were likely fed or grazed together around the site. In general, sheep and goat are often kept together in mixed herds, although sheep are grazers, while goats are more flexible feeders and will browse more [101]. The general diet was mainly composed of C_3_ vegetation, but some individuals show significant consumption of C_4_ or marine plants like the cattle, and this occurred during all periods.

Similar ^13^C-enriched results were also found for Middle and Late Bronze Age sheep/goats from Ya’amūn, Jordan [102]. These were attributed to substantial amounts of C_4_ plants in their diet, possibly due to transhumance in arid and semi-arid locations away from the site during the rainy season. The δ^15^N results of the sheep and goats are nearly identical for all periods except for the Middle Bronze Age where the sheep are elevated over the goats by ~2‰ (Fig 7B), but this difference was not statistically significant (Independent-samples t-test; p = 0.166). While the δ^13^C values are the same for the sheep and goats during the Middle Bronze Age, this difference in δ^15^N values could suggest (in contrast to the other periods) that the sheep and goat were grazed or kept separately around the site, and additional work in terms of tooth serial sections is necessary to examine this possibility in more detail (e.g., [103, 104]).

### Diachronic wild animal isotopic patterns

#### Suids

With a total of 114 suid bones identified (14 wild boar (*Sus scrofa*), 7 domesticated pigs (*Sus scrofa* f. domestica), 93 wild or domesticated), the numerical importance of pigs is negligible compared to that of the domesticated sheep, goat and cattle. They represent less than 1% of the identified large mammals (Linseele et al. [44]: 432). At Tell Tweini, it appears that the number of suids are highest during the Early Bronze Age and that their numbers decrease through time (Linseele et al. [44]: 432). This may suggest that over time domestic pig became less abundant and wild boar relatively more frequent. It was shown that the importance of pigs varied with time in the Levant, and that it was particularly low during the Late Bronze Age. The underlying causes are complex and likely a combination of various environmental, political, economic and cultural reasons [105, 106]. A diachronic analysis of the pigs is hampered by the relatively small number of samples and in particular by the fact that it was difficult to distinguish the domestic and wild form. In general, the suids have ^13^C-depleted values compared to the domestic animals and are similar to the deer (Fig 7A). In the Early Bronze Age, the suids have relatively high δ^15^N values, which are similar to the Early Bronze Age dog (Fig 7B). Suids then show a progressive decrease in δ^15^N results during the Middle and Late Bronze Age. It is not excluded that this might reflect a transition towards more wild boar hunting versus pig keeping, with the pigs having more access to human food waste and thus showing higher δ^15^N values in the Early Bronze Age.

#### Deer

The most common game animal found at Tell Tweini is the fallow deer. The small number of specimens analyzed makes it difficult to diachronically compare the Early and Middle Bronze Age periods. The fallow deer have ^13^C-depleted results during all time periods, and these are similar to the suids (Fig 7A). There is little change between the mean δ^13^C values (0.3‰) of the fallow deer between the Late Bronze Age and the Iron Age. In terms of δ^15^N values, fallow deer have similar results during all periods, and these are also the lowest of all the domestic and wild animals studied (Fig 7B). These ^13^C- and ^15^N-depleted results are commonly found in archaeologically deer from a variety of contexts across the globe, and can be used to reconstruct the environmental isotopic baseline of a site (e.g., [8, 107–109]).

#### Middle Bronze Age human diet: Why such low δ^15^N values around the Mediterranean?

Individuals with low δ^15^N values have also been reported for other Bronze Age populations in the Eastern Mediterranean. At the site of Sidon in Lebanon, Schutkowski and Ogden [110] found that adult Middle Bronze Age humans had δ^15^N values that ranged from 5.2‰ to 11.0‰ with a mean of 8.6±1.3‰. At the Greek Middle Bronze Age sites of Lerna and Asine some individuals have δ^15^N values between 7–9‰ [111, 112]. In addition, this trend of humans with low δ^15^N values carries over into the Late Bronze Age. In Greece, Petroutsa and Manolis [113] studied four Late Bronze age sites and many of the individuals had δ^15^N results <7.0‰, especially at the site of Aghia Triada (δ^15^N = 7.2±0.5‰). Furthermore, Middle and Late Bronze Age humans from Ya’amūn, Jordan had δ^15^N results that were similar or slightly elevated to those of the sheep/goats [102]. These results were argued to represent significant consumption of ^15^N-depleted pulses which reduced the human δ^15^N results and masked the true consumption of animal protein in the population. Finally, domestic and wild animals were found to represent minor components for human diets at the Bronze Age sites of Archontiko and Thessaloniki Toumba, Greece, where cereal crops were determined to be the main sources of consumed protein [8].

At Tell Tweini, the Middle Bronze Age humans consumed an exclusive terrestrial C_3_ diet devoid of isotopically detectable amounts of C_4_, freshwater or marine protein. Interestingly, these individuals had unusually low δ^15^N results which were similar or only slightly elevated compared to the domestic food animals (cattle, sheep, goat), and even lower than the dogs from the same period (Fig 3). This suggests a predominantly plant-based diet that was centered on wheat, pulses and olives and to a lesser extent, grapes. These isotopic results agree with the main archaeobotanical finds recovered from the burials (leguminous crops, olives, grapes and figs) [84]. In particular, one of these graves contained a jar with a substantial offering of bitter vetch seeds (~600 g), emphasizing a certain importance of this pulse crop in the human diet. Further, excavation of the ovens at Tell Tweini revealed high amounts of grape seeds and olive pits which alsoattests to the importance of these foods to the human diet [84]. A carbohydrate rich diet is also supported by the high frequency of dental caries found in these Middle Bronze Age individuals [46].

However, these individuals were not vegetarians, and the diet was certainly supplemented with meat or secondary products from domestic animals. While many Middle Bronze Age burials produced no faunal offerings, one grave contained the skull of a sheep/goat that was placed at the feet of the individual [84]. This indicates the sheep and goats were consumed, likely during special occasions such as funerary rites. Data complied on the age at death for the sheep and goats also indicates that secondary products were used (milk) since most animals were slaughtered after the age of four years [44]. Apart from the sheep and goats, cattle must have served as food for the human population during all periods of occupation at Tell Tweini. This can be deduced from the isotopic results of the dogs as well as the fragmentary and disarticulated nature of the cattle skeletal remains, which included the presence of butchery marks [44]. In terms of numbers of bones, cattle are less frequently found than caprines, but considering the much larger live weight of cattle, they must nevertheless have provided a significant quantity of the meat when consumed at Tell Tweini. Cattle of different age categories are present, but based on the presence of heavily worn teeth, the majority of the animals were adult. This suggests the cattle were used for milk as well as for meat in addition to being draft animals.

Finally, a large variety of fish were recovered at Tell Tweini during all periods and isotopically analyzed [15], but these account for only a small number of the total faunal remains [44] and no detectable amounts of marine protein were identified in the Middle Bronze Age humans. It is surprising that there is a significant lack of fish consumption throughout the Eastern Mediterranean during these periods in the isotopic record (e.g. [113, 114]). This may reflect a real finding or the problem that the amount of fish consumption was too low to be detected with bulk stable isotope ratio values [115], and alternative techniques such as compound-specific isotope analysis of amino acids may be able to provide more detailed dietary information (e.g. [116, 117]).

At Tell Tweini, it is also possible the high consumption of pulses such as bitter vetch lowered the Middle Bronze Age human δ^15^N results and thereby obscured the true level of consumption of animal protein. However, if these humans were eating a diet high in both animal protein and pulses, higher bone collagen δ^15^N values might be expected given the high protein content of both lentils and meat protein. Further, a diet with a higher amount of animal protein should result in ^13^C-enriched values as well as additional isotopic scatter in the humans, mirroring the highly variable δ^13^C results of the domestic animals. As this is not the case, it appears likely the human diet was more centered on plant proteins, and that a substantial amount of protein was derived from the bitter vetch as well as other crops. Curiously, these human δ^13^C results are nearly all identical, except for a single individual, which is especially surprising given the wide range of isotopic values of the available foods during the Middle Bronze Age. The reasons for this remain unknown but could suggest some sort of processed or refined communal diet was consumed by these individuals. Summarizing, the diet during the Middle Bronze Age at Tell Tweini, appears comparable to what is considered today a typical Mediterranean diet with bread (wheat), olives, pulses, likely some cheese and milk and small amounts of meat. This is in agreement with the vast majority of isotopic evidence from the Eastern Mediterranean and points to the fact that this ‘modern Mediterranean diet’ has been practiced in the region since at least the Bronze Age.

#### Agricultural production within the extended environmental and cultural framework of Tell Tweini

The agricultural production at Bronze and Iron Age Tell Tweini requires consideration against the background of two deeply entangled narratives of the Eastern Mediterranean history of humankind, i.e., the major climate shifts of the Meghalayan era of the Holocene [22–27, 29, 31–34] and the cross-cultural relations of that time (e.g., [35]), as visible in the archaeological and historical evidence of trade networks and warfare. Various climate-related conclusions have been drawn about ancient agriculture in relation with the Holocene climatic events around 4.2 and 3.2 ka BP by different researchers [4, 9, 26, 27, 29, 31, 33, 55, 67, 75, 78, 83, 92, 118–120]. Despite of having been accused of environmental determinism, most of these researchers are well aware of the ability of ancient societies to counteract environmental threats [121–123]. For example, adaptations of cereal producing societies to changing climatic conditions include regional abandonment and habitat-tracking to riparian refugia at the onset of the 4.2 ka BP event and political state formation, increasing and enhancing surplus agro-production and politico-territorial expansion as a reaction to decreasing aridity around 3.9 ka BP [124]. From an emic perspective, we may classify all these actions to be part of agricultural niche construction, however, they likely were locally very variable, depending on regional landscape and societal differences.

Geoarchaeological research around Tell Tweini detected the existence of wetlands until around 1000 BC, which likely buffered against climatic fluctuations and their local effects during Cold Periods 3 and 4 [27–29]. Although the Early Bronze Age archaeobotanical record from Tell Tweini is limited, it can be noted that wetland habitats are reflected in the wild plant seed assemblages throughout all periods, and together with a generally advantageous location close to the coast the precipitation regime was likely generally higher compared to more inland regions [44]. Drought stress in the Early and Middle Bronze Ages is not observed in the mean Δ^13^C values of the cereals and stress signals occur in only very few grains (see also S3 and S4 Figs in S1 File). Comparing barley from Tell Tweini to a supra-regional data set [12], it appears to have been better watered than most of the other Near Eastern settlements.

The relatively arid sequence between 1200 and 900 BC (3150–2900 cal. BP) has often been related to crop failure, political upheaval and economic collapse co-determining social stress and famine across the Levantine and Mesopotamian regions (e.g., [35, 118]). Most of these studies have in common that they are based on data not directly deduced from agricultural remains, such as taxonomic and isotopic evidence from ancient crop and faunal assemblages. Pollen-based climate reconstructions of the mid-late Holocene also frequently suggest climate-dependent food availability (e.g., [31, 119, 120]). For example, Cold Periods 3 and 4 are characterized as the sequences with the highest intensity of ecological change and have been interpreted as having impacted food security, based on very low cereal pollen accumulation rates [29]. Such models assume that pollen indicators of crop cultivation and arboriculture represent an indirect proxy of food availability, and that drought phases indicate periods of low crop production [29]. However, methodological issues might hamper the reliability of such unilateral arguments. Primarily, differences in pollination and pollen transfer are problematic, i.e., the cleistogamous cereal pollen tends to be underrepresented in a pollen profile from alluvial deposits, which are generally assumed to reflect the catchment area of the sediment-transporting river [125]. While a relation between local climate effects and crop yields is self-evident, human actors can modify the absolute crop yield and buffer crop failure considerably by diverse measures [126]. Therefore, the actual remains of harvested crops in ancient settlements are a better indicator of food availability, making archaeobotanical studies indispensable proxies for investigating ancient agricultural food production.

According to the geoarchaeological record, the wetland around Tell Tweini persisted throughout the Late Bronze Age [28], whereas for the Early Iron Age I (1200–900 BC) a ‘warm steppe/hot desert biome’ was reconstructed [27, 29], a time that is marked by the 3.2 kyr BP event and historically and archaeologically documented societal upheaval and collapse in the whole Levant, not least reflected in the fall of the Ugarit Kingdom, inclusive of Gibala/Tell Tweini, shortly after 1200 BC, and the later Iron Age I destruction level at Tell Tweini ca. 1050 BC [37]. Nevertheless, settlement at Tell Tweini continues with the beginning of Iron Age II and has preserved archaeological evidence of olive oil production [20]. The Late Bronze and Iron Age Δ^13^C record of all crops at Tell Tweini indicates a high level of water availability for all crop species, when compared to other sites (for barley see [12], for grape and olive S5 Fig in S1 File). The only exception to this rule is the Iron Age Δ^13^C values for grape, which seem to be stressed compared to the previous periods (Fig 6A). This phenomenon might be related to the fact that we combined Iron Age I and II samples, but Iron Age grape pips were only available from Iron Age I, which falls into Cold Period 3, associated with the 3.2 ka BP event. This means that we might expect for some of the other crops slightly lower moisture availability in the Iron Age I, which might be superimposed by higher moisture availability of the Iron Age II samples. Notwithstanding, considering the lowest absolute mean Δ^13^C values of any of the crops at Tell Tweini and comparing them with those at other contemporary sites in the wider region, we might conclude that the inhabitants of Tell Tweini handled the increased aridity during this period very well, and in most cases even better than at other contemporaneous settlements.

While the multiplicity of decision making in food production by human societies is well-known [127, 128], the interplay of different variables of inner- and interregional differences in the local environmental capacity of a given area (determined by rainfall, soil depth, various hydrological conditions and topographic indices such as slope and elevation) and individual/cultural preferences seems to be best understood by looking at the local evidence. This is probably the main reason why, despite recognizable environmental effects on food production, attempts of generalizations over wider spatial and temporal units proved invalid [79, 129, 130]. In particular, the role of ‘Rapid Climate Change’ events on human societies, while potentially having devastating effects on short-term production capacity, is regionally unclear [131]. In a similar vein, collapse and destruction of settlements, willingly attributed to natural catastrophes or martial invasions require local re-evaluation [132]. Other studies suggest that populations became increasingly disconnected from climate fluctuations over the course of the Mid-Late Holocene even though absolute numbers of people increased [133–135]. Furthermore, risk-buffering may have played a larger role in ancient food production strategies than so far recognized [126]. The current evidence from Tell Tweini indicates that differences in regional and local climatic effects and human actions ensuring sustainable yields require an adjustment of the generalized models by additional proxies, such as geoarchaeological, archaeobotanical, faunal and stable isotopes addressing agricultural niche construction processes at specific locations.

The extraordinary structural features of Tell Tweini’s location, such as its strategic position at the junction of two rivers, about 1.5 km from the Mediterranean coast, its geographic and commercial association to Late Bronze Age II Ugarit as well as numerous archaeological findings of geographically distant origin, such as Mesopotamian cylinder seals, Late Helladic and Cypriot pottery or the shared loom weight typology with Cyprus and the Philistine settlements of the southern Levant [17, 30] suppose that Tell Tweini played a major role in the Levantine economy of the Bronze and Iron Age, which included a strong agricultural output for their own population as well as for possible participation in trading. Thus, by viewing past agricultural production at Tell Tweini into the wider spatial context we can summarize that the ancient inhabitants experienced baseline conditions that were generally more advantageous than at settlements more inland and at locations that were further away from wetland ecosystems, and that allowed, as indicated by the low stress level of the plants, surplus production. Given that olive oil production played a role in everyday life, we can conclude from the Δ^13^C values in olives, indicating a higher water availability than at other sites at any time, that olive oil production may have potentially exceeded or at least have been comparable to the major centers of oil production during the Bronze and Iron Age, and even withstood climatic aridification processes.

## Conclusions

Our stable isotope ratio analyses of human, faunal, and archaeobotanical remains from Tell Tweini have allowed a diachronic study of the development of agriculture and animal husbandry in the Eastern Mediterranean, and to place it in a broader geographical context by comparison with other Bronze Age and Iron Age sites. It was also possible to analyze the human diet of individuals from Middle Bronze Age II. Consistent with the generally sustainable landscape features of extensive wetlands, the Δ^13^C measurements on plant species revealed that emmer and free threshing wheat, olives, bitter vetch, rye grass, and barley were adequately or well-watered during all periods of occupation, with some probably negligible aridity trends for free-threshing wheat and barley during the Middle Bronze Age. Grapes had the most elevated Δ^13^C results, and this suggests that they were likely very well cared for and experienced excellent growing conditions, which reflects the general Levantine emphasis on grapevine production during the Bronze and Iron Ages. However, additional research on water availability and how it impacts grape Δ^13^C results is needed to fully validate these conclusions. The δ^15^N results from the archaeobotanical remains revealed that animal manure was used as a soil fertilizer in different amounts for different crops. For nearly all periods, manuring was likely applied in a low to medium amount as many of the plant δ^15^N results were in the range of 3–6‰ and overlap with the δ^15^N values of the domestic food animals. During the Iron Age nitrogen levels are increased for all crops, excluding emmer wheat, thus implying either higher amounts of manure and/or extension of fields into previously uncultivated areas with still high natural soil fertility.

In general, the domestic animals (cattle, sheep, goats) consumed dominantly C_3_ terrestrial diets and were found to isotopically cluster, which is evidence they were kept together or grazed in similar environments. However, some cattle, sheep and goats also fed on mixed C_3_ and C_4_ or marine plants. This potential C_4_ dietary signal appears to originate from the wild plant assemblage, which includes some C_4_ species, such as salt tolerant grasses and shrubs from coastal and wetland marshes or from anthropogenic habitats which surrounded Tell Tweini. Additional isotopic measurements (e.g., δ^13^C_AA_ analysis; [63]) are needed to fully resolve this question. The Middle Bronze Age humans all were found to consume a homogenous C_3_ terrestrial diet that had no measurable input from C_4_, freshwater or marine protein sources. Their diet was relatively low in animal protein and based on wheat, pulses and olives and to a lesser extent, grapes, comparable to other Mediterranean sites of the wider region.

In view of the known critical factors influencing Bronze and Iron Age agriculture in the Eastern Mediterranean region, such as the global climate fluctuations at the end of the Early and Late Bronze Age or the collapse of the socio-economic system in connection with migrations, at least in part of a warlike nature, which are described as the invasion of the "Sea Peoples", agricultural production at Tell Tweini proves to be comparatively resilient. Thus, despite the destruction of Tell Tweini in the first quarter of the 12^th^ century BC, a revival of urban life and trading systems in the 11^th^ century BC and continuing into the Iron Age II is evident. Olive oil and wine production in particular took place under excellent conditions, as evidenced by the good nitrogen supply of the soil through moderate fertilization and sufficient water availability, perhaps even selective irrigation. The strong economic integration into the Mediterranean trade system, especially in the Late Bronze Age and Iron Age, is evident in comparison to other sites in the region, which were more negatively affected by climatic fluctuations due to their more southerly or easterly geographical location (e.g. Tel Kabri, Tell Mozan). Finally, phenomenologically intriguing is the observation that Ugarit, situated ca. 35 km to the north, once the capital of the empire, witnessed negligible resettlement subsequent to its Late Bronze Age devastation. The inquiry into whether less favourable environmental conditions or the absence of adaptive strategies among its inhabitants played a pivotal role, relative to Tell Tweini, warrants further investigation.

## Supporting information

S1 Checklist Inclusivity in global research. (DOCX)

S1 File (DOCX)

S1 Data (XLSX)

## References

[1] Lee-Thorp JA. On isotopes and old bones. Archaeometry. 2008;50:925–50. doi: 10.1111/j.1475-4754.2008.00441.x WOS:000261215800002.

[2] Reitsema LJ. Laboratory and field methods for stable isotope analysis in human biology. Am J Hum Biol. 2015;27(5):593–604. Epub 20150722. doi: 10.1002/ajhb.22754 . 26202876

[3] Fiorentino G, Ferrio JP, Bogaard A, Araus JL, Riehl S. Stable isotopes in archaeobotanical research. Veget Hist Archaeobot. 2015;24(1):215–27.

[4] Ferrio J, Alonso N, Voltas J, Araus J. Grain weight changes over time in ancient cereal crops: Potential roles of climate and genetic improvement. Journal of Cereal Science. 2006;44(3):323–32. doi: 10.1016/j.jcs.2006.07.013

[5] Ferrio JP, Aguilera M, Voltas J, Araus JL. Chapter Three—Stable carbon isotopes in archaeological plant remains. In: Montenari M, editor. Stratigraphy & Timescales. 5: Academic Press; 2020. p. 107–45.

[6] Richards MP. Isotope Analysis for Diet Studies. In: Britton K, Richards MP, editors. Archaeological Science: An Introduction. Cambridge: Cambridge University Press; 2020. p. 125–44.

[7] Styring AK, Fraser RA, Arbogast R-M, Halstead P, Isaakidou V, Pearson JA, et al. Refining human palaeodietary reconstruction using amino acid δ15N values of plants, animals and humans. Journal of Archaeological Science. 2015;53:504–15. doi: 10.1016/j.jas.2014.11.009

[8] Nitsch E, Andreou S, Creuzieux A, Gardeisen A, Halstead P, Isaakidou V, et al. A bottom-up view of food surplus: using stable carbon and nitrogen isotope analysis to investigate agricultural strategies and diet at Bronze Age Archontiko and Thessaloniki Toumba, northern Greece. World Archaeology. 2017;49(1):105–37. doi: 10.1080/00438243.2016.1271745

[9] Fiorentino G, Caracuta V, Calcagnile L, D’Elia M, Matthiae P, Mavelli F, et al. Third millennium B.C. climate change in Syria highlighted by Carbon stable isotope analysis of 14C-AMS dated plant remains from Ebla. Palaeogeography, Palaeoclimatology, Palaeoecology. 2008;266(1):51–8. doi: 10.1016/j.palaeo.2008.03.034

[10] van der Plicht J, Akkermans PMMG, Buitenhuis H, Kaneda A, Nieuwenhuyse O, Russell A. Tell Sabi Abyad, Syria: An Interpretation of Stable Isotope Values of Faunal Bone Collagen. Radiocarbon. 2012;54(3–4):281–9. Epub 2016/07/18. doi: 10.1017/S003382220004707X

[11] Tomczyk J, Szostek K, Komarnitki I, Mańkowska-Pliszka H, Zalewska M. Dental caries and chemical analyses in reconstruction of diet, health and hygienic behaviour in the Middle Euphrates valley (Syria). Arch Oral Biol. 2013;58(6):740–51. Epub 20130123. doi: 10.1016/j.archoralbio.2012.12.014 . 23352446

[12] Riehl S, Pustovoytov KE, Weippert H, Klett S, Hole F. Drought stress variability in ancient Near Eastern agricultural systems evidenced by δ13C in barley grain. Proceedings of the National Academy of Sciences. 2014;111(34):12348–53. doi: 10.1073/pnas.1409516111 25114225 PMC4151733

[13] Sołtysiak A, Schutkowski H. Continuity and change in subsistence at Tell Barri, NE Syria. Journal of Archaeological Science: Reports. 2015;2:176–85. doi: 10.1016/j.jasrep.2015.01.011

[14] Sołtysiak A, Schutkowski H. Stable isotopic evidence for land use patterns in the Middle Euphrates Valley, Syria. American Journal of Physical Anthropology. 2018;166(4):861–74. doi: 10.1002/ajpa.23480 29665014

[15] Fuller BT, Van Neer W, Linseele V, De Cupere B, Chahoud J, Richards MP. Fish δ13C and δ15N results from two Bronze/Iron Age sites (Tell Tweini & Sidon) along the Levantine coast. Journal of Archaeological Science: Reports. 2020;29:102066. doi: 10.1016/j.jasrep.2019.102066

[16] Kharobi A, Stantis C, Maaranen N, Schutkowski H. Once were warriors: Challenging occupation preconceptions in Lebanese weapon-associated burials (Middle Bronze Age, Sidon). International Journal of Osteoarchaeology. 2021;31(6):1155–68. doi: 10.1002/oa.3027

[17] Bretschneider J, Jans G. About Tell Tweini (Syria): Artefacts, Ecofacts and Landscape. Leuven: Peeters publishers; 2019.

[18] van Soldt WH. Studies in the topography of Ugarit (2). The borders of Ugarit. Ugarit Forschungen 1997;30:703–44.

[19] Akkermans PMMG, Schwartz GM. The archaeology of Syria: from complex hunter-gatherers to early urban societies (c. 16,000–300 BC). Cambridge: Cambridge University Press; 2004.

[20] Bretschneider J, Van Vyve A-S, Jans G, editors. Tell Tweini: A Multi-Period Harbour Town at the Syrian Coast. International Conference on the Relations of Egypt and the Near East in the Bronze Age; 2011 2010-09-01; Prague, Czech Republic: Charles University.

[21] Bar-Matthews M, Ayalon A, Kaufman A. Middle to Late Holocene (6,500 Yr. Period) Paleoclimate in the Eastern Mediterranean Region from Stable Isotopic Composition of Speleothems from Soreq Cave, Israel. In: Issar AS, Brown N, editors. Water, Environment and Society in Times of Climatic Change: Contributions from an International Workshop within the framework of International Hydrological Program (IHP) UNESCO, held at Ben-Gurion University, Sede Boker, Israel from 7–12 July 1996. Dordrecht: Springer Netherlands; 1998. p. 203–14.

[22] Cullen HM, deMenocal PB, Hemming S, Hemming G, Brown FH, Guilderson T, et al. Climate change and the collapse of the Akkadian empire: Evidence from the deep sea. Geology. 2000;28(4):379–82. doi: 10.1130/0091-7613(2000)28&lt;379:Ccatco&gt;2.0.Co;2

[23] Staubwasser M, Sirocko F, Grootes PM, Segl M. Climate change at the 4.2 ka BP termination of the Indus valley civilization and Holocene south Asian monsoon variability. Geophysical Research Letters. 2003;30(8). doi: 10.1029/2002GL016822

[24] Hazan N, Stein M, Agnon A, Marco S, Nadel D, Negendank JFW, et al. The late Quaternary limnological history of Lake Kinneret (Sea of Galilee), Israel. Quaternary Research. 2005;63(1):60–77. doi: 10.1016/j.yqres.2004.09.004

[25] Migowski C, Stein M, Prasad S, Negendank JFW, Agnon A. Holocene climate variability and cultural evolution in the Near East from the Dead Sea sedimentary record. Quaternary Research. 2006;66(3):421–31. doi: 10.1016/j.yqres.2006.06.010

[26] Kaniewski D, Van Campo E, Weiss H. Drought is a recurring challenge in the Middle East. Proceedings of the National Academy of Sciences. 2012;109(10):3862–7. doi: 10.1073/pnas.1116304109 22355126 PMC3309751

[27] Kaniewski D, Paulissen E, Van Campo E, Al-Maqdissi M, Bretschneider J, Van Lerberghe K. Middle East coastal ecosystem response to middle-to-late Holocene abrupt climate changes. Proceedings of the National Academy of Sciences. 2008;105(37):13941–6. doi: 10.1073/pnas.0803533105 18772385 PMC2544558

[28] Baeteman C, Bogemans F. In Search of a Harbour in the Past Landscapes of Tell Tweini. Identification of Sedimentary Environments in Support of an Archaeological Issue. In: Bretschneider J, Jans G, editors. About Tell Tweini (Syria): Artefacts, Ecofacts and Landscape. Research Results of the Belgian Mission, Orientalia Lovaniensia Analecta Series 281. Leuven: Peeters publishers; 2019. p. 619–37.

[29] Kaniewski D, Marriner N, Cheddadi R, Fischer P, Otto T, Luce F, et al. Climate Change and Social Unrest: A 6,000‐Year Chronicle From the Eastern Mediterranean. Geophysical Research Letters. 2020;47:e2020GL087496. doi: 10.1029/2020GL087496

[30] Bretschneider J, Jans G, Van Vyve A-S, Hameeuw H, Vansteenhuyse K. TELL TWEINI: A LONG STORY SHORT. In: Bretschneider J, Jans G, editors. About Tell Tweini (Syria): artefacts, ecofacts and landscape: research results of the Belgian Mission. Orientalia Lovaniensia Analecta. 281. Leuven: Peeters Publishers; 2019. p. 1–30.

[31] Kaniewski D, Paulissen E, Van Campo E, Weiss H, Otto T, Bretschneider J, et al. Late second–early first millennium BC abrupt climate changes in coastal Syria and their possible significance for the history of the Eastern Mediterranean. Quaternary Research. 2010;74(2):207–15. doi: 10.1016/j.yqres.2010.07.010

[32] Kaniewski D, Van Campo E, Van Lerberghe K, Boiy T, Vansteenhuyse K, Jans G, et al. The Sea Peoples, from Cuneiform Tablets to Carbon Dating. PLOS ONE. 2011;6(6):e20232. doi: 10.1371/journal.pone.0020232 21687714 PMC3110627

[33] Kaniewski D, Marriner N, Cheddadi R, Morhange C, Bretschneider J, Jans G, et al. Cold and dry outbreaks in the eastern Mediterranean 3200 years ago. Geology. 2019;47(10):933–7. doi: 10.1130/g46491.1

[34] Jung R. Push and Pull Factors of the Sea Peoples between Italy and the Levant. In: Driessen J, editor. The Archaeology of Forced Migration Crisis-induced mobility and the Collapse of the 13th c BCE Eastern Mediterranean (Aegis 15). Louvain2018. p. 279–312.

[35] Cline EH. 1177 B.C.: The Year Civilization Collapsed. REV—Revised ed: Princeton University Press; 2021.

[36] Vansteenhuyse K, editor The Bronze to Iron Age Transition at Tell Tweini (Syria)2010.

[37] Bretschneider J, Jans G, Van Vyve A-S, Debruyne M. “The ‘ochre’ Room: Shedding Some Light on a ‘Dark’ Period of Transition: Tell Tweini in the Early Iron Age. In: Boiy T, Bretschneider J, Goddeeris A, Hameeuw H, Jans G, Tavernier J, editors. The ancient near east, a life! Festschrift Karel Van Lerberghe. Orientalia Lovaniensia Analecta. 220. Leuven: Peeters; 2012. p. 59–74.

[38] Bretschneider J, Jans G, Van Vyve A. Les campagnes des Fouilles de 2009 et 2010. In: Al-Maqdissi M, Van Lerberghe K, Bretschneider J, Badawi M, editors. Tell Tweini: onze campagnes de fouilles syro-belges (1999–2010). Damas: Ministère de la Culture; 2010. p. 131–46.

[39] Bretschneider J, Van Lerberghe K. Tell Tweini, Ancient Gibala, between 2600 B.C.E. and 333 B.C.E. In: Bretschneider J, Van Lerberghe K, editors. In search of Gibala An archaeological and historical study based on eight seasons of excavations at Tell Tweini (1999–2007) in the A and C fields. Barcelona: Editorial Ausa; 2008. p. 11–68.

[40] Cappers R, Neef R, Bekker R. Digital Atlas of Economic Plants. 9 ed. Eelde: Barkhuis Publishing; 2009.

[41] Cappers R, Bekker R, Jans JEA. Digital seed atlas of the Netherlands (2nd Edition). Groningen: Barkhuis Publishing & Groningen University Library; 2006. 502 p.

[42] Nesbitt M. Identification Guide for Near Eastern Grass Seeds. London: University College London; 2006.

[43] Van Zeist W, Bakker-Heeres J. Archaeobotanical studies in the Levant I. Neolithic sites in the Damascus Basin: Aswad, Ghoraife, Ramad. Praehistoria. 1982;24:165–256.

[44] Linseele V, Marinova E, De Cupere B, van der Valk J, Vandorpe P, Van Neer W. Bronze and Iron Age Palaeo-Economy in a Changing Environment. The Bioarchaeology of Tell Tweini, on the Northern Levantine Coast. In: Bretschneider J, Jans G, editors. About Tell Tweini (Syria): Artefacts, Ecofacts and Landscape. Research Results of the Belgian Mission, Orientalia Lovaniensia Analecta Series 281. Leuven: Peeters publishers; 2019. p. 417–617.

[45] Vansteenhuyse K. The ceramic material from Field A (Tell Tweini, Syria). In: Bretschneider J, Van Lerberghe K, editors. In search of Gibala, An archaeological and historical study based on eight seasons of excavations at Tel Tweini (Syria) in the A and C fields (1999–2007). Barcelona: Editorial Ausa; 2008. p. 96–135.

[46] Ricaut F-X. Human remains from a Middle Bronze Age population from Tell Tweini, Syria: preliminary results of the anthropological study. In: Bretschneider J, Van Lerberghe K, editors. In search of Gibala, An archaeological and historical study based on eight seasons of excavations at Tel Tweini (Syria) in the A and C fields (1999–2007). Barcelona: Editorial Ausa; 2008. p. 87–101.

[47] Hameeuw H, Jans G. Burial customs at Tell Tweini–Field A. In: Bretschneider J, Van Lerberghe K, editors. In search of Gibala, An archaeological and historical study based on eight seasons of excavations at Tel Tweini (Syria) in the A and C fields (1999–2007). Barcelona: Editorial Ausa; 2008. p. 75–86.

[48] Jans G, Bretschneider J. A Collective Middle Bronze Age II Tomb at Tell Tweini Field A. In: Bretschneider J, Jans G, editors. About Tell Tweini (Syria): Artefacts, Ecofacts and Landscape Research Results of the Belgian Mission (Orientalia Lovaniensia Analecta Series 281) 2019. p. 197–238.

[49] Richards MP, Hedges REM. Stable Isotope Evidence for Similarities in the Types of Marine Foods Used by Late Mesolithic Humans at Sites Along the Atlantic Coast of Europe. Journal of Archaeological Science. 1999;26(6):717–22. doi: 10.1006/jasc.1998.0387

[50] Brown TA, Nelson DE, Vogel JS, Southon JR. Improved collagen extraction by modified Longin method. Radiocarbon. 1988;30(2):171–7.

[51] Vaiglova P, Snoeck C, Nitsch E, Bogaard A, Lee-Thorp J. Impact of contamination and pre-treatment on stable carbon and nitrogen isotopic composition of charred plant remains. Rapid Communications in Mass Spectrometry. 2014;28(23):2497–510. doi: 10.1002/rcm.7044 25366397 PMC4403960

[52] Schwarcz HP, Schoeninger MJ. Stable isotope analyses in human nutritional ecology. American Journal of Physical Anthropology. 1991;34(S13):283–321. doi: 10.1002/ajpa.1330340613

[53] Riehl S. Stable Isotopes in Ancient Agriculture. A Companion to Ancient Agriculture 2020. p. 55–81.

[54] Nitsch EK, Charles M, Bogaard A. Calculating a statistically robust δ13C and δ15N offset for charred cereal and pulse seeds. STAR: Science & Technology of Archaeological Research. 2015;1(1):1–8. doi: 10.1179/2054892315Y.0000000001

[55] Ferrio JP, Araus JL, Buxó R, Voltas J, Bort J. Water management practices and climate in ancient agriculture: inferences from the stable isotope composition of archaeobotanical remains. Veget Hist Archaeobot. 2005;14(4):510–7. doi: 10.1007/s00334-005-0062-2

[56] Ferrio J, Voltas J, Araus J. A smoothed curve of d13C of atmospheric CO2 from 16,100 BCE to 2010 CE 2012 [cited February 10, 2021.]. Available from: http://web.udl.es/usuaris/x3845331/AIRCO2_LOESS.xls.

[57] DeNiro MJ. Postmortem preservation and alteration of in vivo bone collagen isotope ratios in relation to palaeodietary reconstruction. Nature. 1985;317(6040):806–9.

[58] Ambrose SH. Preparation and characterization of bone and tooth collagen for isotopic analysis. Journal of Archaeological Science. 1990;17(4):431–51. doi: 10.1016/0305-4403(90)90007-R

[59] Smith CI, Craig OE, Prigodich RV, Nielsen-Marsh CM, Jans MME, Vermeer C, et al. Diagenesis and survival of osteocalcin in archaeological bone. Journal of Archaeological Science. 2005;32(1):105–13. doi: 10.1016/j.jas.2004.07.003

[60] Benecke N. Der Mensch und seine Haustiere. Die Geschichte einer jahrtausendenalten Beziehung. Stuttgart 1994.

[61] Fuller BT, Molleson TI, Harris DA, Gilmour LT, Hedges REM. Isotopic Evidence for Breastfeeding and Possible Adult Dietary Differences from Late/Sub-Roman Britain. 2006;129(1):45–54. doi: 10.1002/ajpa.20244 16229026

[62] Cunningham PL. Plants included in the diet of Arabian Sand Gazelle (Reem) from Saudi Arabia. Journal of King Saud University—Science. 2013;25(2):167–73. doi: 10.1016/j.jksus.2012.10.002

[63] Ma Y, Grimes V, Van Biesen G, Shi L, Chen K, Mannino MA, et al. Aminoisoscapes and palaeodiet reconstruction: New perspectives on millet-based diets in China using amino acid δ13C values. Journal of Archaeological Science. 2021;125:105289. doi: 10.1016/j.jas.2020.105289

[64] Bocherens H, Argant A, Argant J, Billiou D, Crégut-Bonnoure E, Donat-Ayache B, et al. Diet reconstruction of ancient brown bears (Ursus arctos) from Mont Ventoux (France) using bone collagen stable isotope biogeochemistry (13C, 15N). Canadian Journal of Zoology. 2004;82(4):576–86. doi: 10.1139/z04-017

[65] Zohary M. Geobotanical Foundations of the Middle East, vol 1. Stuttgart: Gustav Fisher Verlag; 1973.

[66] Farquhar GD, Ehleringer JR, Hubick KT. Carbon Isotope Discrimination and Photosynthesis. Annual Review of Plant Physiology and Plant Molecular Biology. 1989;40(1):503–37. doi: 10.1146/annurev.pp.40.060189.002443

[67] Riehl S. Archaeobotanical evidence for the interrelationship of agricultural decision-making and climate change in the ancient Near East. Quaternary International. 2009;197(1):93–114. doi: 10.1016/j.quaint.2007.08.005

[68] Nesbitt M, Samuel D, editors. From staple crop to extinction? The archaeology and history of the hulled wheats. Proceedings of the First International Workshop on Hulled Wheats: promoting the conservation and use of underutilized and neglected crops; 1996 21–22 July 1995; Castelvecchio Pascoli, Tuscany, Italy: IPGRI (International Plant Genetic Resources Institute).

[69] Riehl S, Nesbitt M. Crops and cultivation in the Iron Age Near East: change or continuity? In: Fischer B, editor. Identifying Changes—The transition from Bronze to Iron2003. p. 301–12.

[70] Riehl S. Plant production at Qatna in the environmental and supra-regional economic context. In: Pfälzner P, editor. Qatna and the networks of Bronze Age globalism; Oktober 2009; Akten einer internationalen Konferenz in Stuttgart 2015.

[71] Nicolì M. Chapter 24 Archaeobotany. In: Cline EH, Yasur-Landau A, Ratzlaff A, editors. Excavations at Tel Kabri III. Culture and History of the Ancient Near East. Leiden, The Netherlands: Brill. 2023.

[72] Riehl S. Chapter 25 Stable Isotope Measurements on Seed Remains. In: Cline E, Yasur-Landau A, Ratzlaff A, editors. Excavations at Tel Kabri III. Leiden, The Netherlands: Brill. 2023.

[73] Craine JM, Elmore AJ, Aidar MPM, Bustamante M, Dawson TE, Hobbie EA, et al. Global patterns of foliar nitrogen isotopes and their relationships with climate, mycorrhizal fungi, foliar nutrient concentrations, and nitrogen availability. New Phytologist. 2009;183(4):980–92. doi: 10.1111/j.1469-8137.2009.02917.x 19563444

[74] Wang C, Wang X, Liu D, Wu H, Lü X, Fang Y, et al. Aridity threshold in controlling ecosystem nitrogen cycling in arid and semi-arid grasslands. Nature Communications. 2014;5(1):4799. doi: 10.1038/ncomms5799 25185641

[75] Styring AK, Charles M, Fantone F, Hald MM, McMahon A, Meadow RH, et al. Isotope evidence for agricultural extensification reveals how the world’s first cities were fed. Nature Plants. 2017;3(6):17076. doi: 10.1038/nplants.2017.76 28581507

[76] Santesteban LG, Miranda C, Barbarin I, Royo JB. Application of the measurement of the natural abundance of stable isotopes in viticulture: a review. Australian Journal of Grape and Wine Research. 2015;21(2):157–67. doi: 10.1111/ajgw.12124

[77] Longobardi F, Casiello G, Centonze V, Catucci L, Agostiano A. Isotope ratio mass spectrometry in combination with chemometrics for characterization of geographical origin and agronomic practices of table grape. Journal of the Science of Food and Agriculture. 2017;97(10):3173–80. doi: 10.1002/jsfa.8161 27885687

[78] Riehl S, Bryson R, Pustovoytov K. Changing growing conditions for crops during the Near Eastern Bronze Age (3000–1200 BC): the stable carbon isotope evidence. Journal of Archaeological Science. 2008;35(4):1011–22. doi: 10.1016/j.jas.2007.07.003

[79] Vermeersch S, Riehl S, Starkovich BM, Kamlah J. Developments in subsistence during the Early Bronze Age through the Iron Age in the southern and central Levant: Integration of faunal and botanical remains using multivariate statistics. Quaternary Science Reviews. 2021;253:106776. doi: 10.1016/j.quascirev.2020.106776

[80] Sorrel P, Mathis M. Mid- to late-Holocene coastal vegetation patterns in Northern Levant (Tell Sukas, Syria): Olive tree cultivation history and climatic change. The Holocene. 2016;26(6):858–73. doi: 10.1177/0959683615622555

[81] Ehrlich Y, Raj H, Mintz E, Regev L, Boaretto E. Olive pits as a high-resolution proxy archive of climate: Δ13C in modern and archaeological olive pits reflecting environmental conditions. Quaternary Science Reviews. 2022;294:107738. doi: 10.1016/j.quascirev.2022.107738

[82] Bretschneider J, Van Lerberghe K. Tell Tweini à travers les millénaires. In: Al-Maqdissi M, Van Lerberghe K, Bretschneider J, Badawi M, editors. Tell Tweini: onze campagnes de fouilles syro-belges (1999–2010). Damas: Ministère de la Culture; 2010. p. 15–76.

[83] Kaniewski D, Paulissen E, Van Campo E, Bakker J, Van Lerberghe K, Waelkens M. Wild or cultivated Olea europaea L. in the eastern Mediterranean during the middle—late Holocene? A pollen-numerical approach. The Holocene. 2009;19(7):1039–47. doi: 10.1177/0959683609341000

[84] Marinova E, Linseele V, Vandorpe P, Van der Valk J. Middle Bronze Age ritual, subsistence and environment at Tell Tweini—Infered from bioarchaeological evidence. In: Boiy T, Bretschneider J, Goddeeris A, Hameeuw H, Jans G, Tavernier J, editors. The ancient Near East, A life! Festschrift Karel Van Lerberghe. Orientalia Lovaniensia Analecta. Leuven: Peeters; 2012. p. 345–64.

[85] Wallace MP, Jones G, Charles M, Fraser R, Heaton THE, Bogaard A. Stable Carbon Isotope Evidence for Neolithic and Bronze Age Crop Water Management in the Eastern Mediterranean and Southwest Asia. PloS one. 2015;10(6):e0127085–e. doi: 10.1371/journal.pone.0127085 . 26061494 PMC4464649

[86] Wallace M, Jones G, Charles M, Fraser R, Halstead P, Heaton THE, et al. Stable carbon isotope analysis as a direct means of inferring crop water status and water management practices. World Archaeology. 2013;45(3):388–409. doi: 10.1080/00438243.2013.821671

[87] Delwiche CC, Zinke PJ, Johnson CM, Virginia RA. Nitrogen Isotope Distribution as a Presumptive Indicator of Nitrogen Fixation. Botanical Gazette. 1979;140:S65—S9.

[88] Van Klinken G, Richards M, Hedges B. An Overview of Causes for Stable Isotopic Variations in Past European Human Populations: Environmental, Ecophysiological, and Cultural Effects. In: Ambrose SH, Katzenberg MA, editors. Biogeochemical Approaches to Paleodietary Analysis. Boston: Springer US; 2002. p. 39–63.

[89] Vaiglova P, Bogaard A, Collins M, Cavanagh W, Mee C, Renard J, et al. An integrated stable isotope study of plants and animals from Kouphovouno, southern Greece: a new look at Neolithic farming. Journal of Archaeological Science. 2014;42:201–15. doi: 10.1016/j.jas.2013.10.023

[90] Ambrose SH. Effects of diet, climate and physiology on nitrogen isotope abundances in terrestrial foodwebs. Journal of Archaeological Science. 1991;18(3):293–317. doi: 10.1016/0305-4403(91)90067-Y

[91] Wang T, Wei D, Chang X, Yu z, Zhang X, Wang C, et al. Tianshanbeilu and the Isotopic Millet Road: Reviewing the late Neolithic/Bronze Age radiation of human millet consumption from north China to Europe. National Science Review. 2019;6(6):1024–39. doi: 10.1093/nsr/nwx015 34691966 PMC8291513

[92] Riehl S, Pustovoytov K, Dornauer A, Sallaberger W. Mid-To-Late Holocene Agricultural System Transformations in the Northern Fertile Crescent: A Review of the Archaeobotanical, Geoarchaeological, and Philological Evidence. In: Giosan L, Fuller D, Nicoll K, Flad RK, Clift P, editors. Climates, Landscapes, and Civilizations. American Geophysical Union monograph series. Washington DC 2012. p. 115–36.

[93] Hrozný F. Das Getreide im alten Babylonien. Ein Beitrag zur Kultur- und Wirtschaftsgeschichte des alten Orients. 1. Vienna: Teil, Hölder; 1913.

[94] Marom N, Bar-Oz G. Zooarcheology and social identity in Bronze and Iron Ages Israel: A research framework. In: De Cupere B LV, Hamilton-Dyer S, editor. Proceedings of the 10th Meeting of ‘ASWA’. Leuven: Peeters Publishing; 2013. p. 227–41.

[95] Gautier A. Taphonomic groups: How and why? Archaeozoologia. 1987;1:47–52.

[96] Pfälzner P. The Elephants of the Orontes. Syria. 2016:159–82.

[97] Massa M, Palmisano A. Change and continuity in the long-distance exchange networks between western/central Anatolia, northern Levant and northern Mesopotamia, c.3200–1600 BCE. Journal of Anthropological Archaeology. 2018;49:65–87. doi: 10.1016/j.jaa.2017.12.003

[98] Guiry EJ. Dogs as Analogs in Stable Isotope-Based Human Paleodietary Reconstructions: A Review and Considerations for Future Use. Journal of Archaeological Method and Theory. 2012;19(3):351–76.

[99] Balasse M, Tresset A, Dobney K, Ambrose SH. The use of isotope ratios to test for seaweed eating in sheep. Journal of Zoology. 2005;266(3):283–91. doi: 10.1017/S0952836905006916

[100] Balasse M, Mainland I, Richards MP. Stable isotope evidence for seasonal consumption of marine seaweed by modern and archaeological sheep in the Orkney archipelago (Scotland). Environmental Archaeology. 2009;14(1):1–14. doi: 10.1179/174963109X400637

[101] Redding R. Decision Making in Subsistence Herding of Sheep and Goats in the Middle East [PhD thesis]: University of Michigan; 1981.

[102] Sandias M, Müldner G. Diet and herding strategies in a changing environment: Stable isotope analysis of Bronze Age and Late Antique skeletal remains from Ya’amūn, Jordan. Journal of Archaeological Science. 2015;63:24–32. doi: 10.1016/j.jas.2015.07.009

[103] Balasse M, Bocherens H, Mariotti A, Ambrose SH. Detection of Dietary Changes by Intra-tooth Carbon and Nitrogen Isotopic Analysis: An Experimental Study of Dentine Collagen of Cattle (Bos taurus). Journal of Archaeological Science. 2001;28(3):235–45. doi: 10.1006/jasc.1999.0535

[104] Frémondeau D, De Cupere B, Evin A, Van Neer W. Diversity in pig husbandry from the Classical-Hellenistic to the Byzantine periods: An integrated dental analysis of Düzen Tepe and Sagalassos assemblages (Turkey). Journal of Archaeological Science: Reports. 2017;11:38–52. doi: 10.1016/j.jasrep.2016.11.030

[105] Vila E. Les restes de suidés, un marqueur archéologique au Levant? De la domestication au tabou: Le cas des suidés au Proche-Orient ancien. Lion MeB, editor. Paris: De Broccard; 2006.

[106] Grigson C, Albarella U, Dobney K, Ervynck A, Rowley-Conwy P. Culture, ecology, and pigs from the 5th to the 3rd millennium Bc around the Fertile Crescent. In: Albarella U, Dobney K, Ervynck A, Rowley-Conwy P, editors. Pigs and Humans: 10,000 Years of Interaction. Oxford: Oxford University Press; 2007. p. 83–108.

[107] Fuller BT, De Cupere B, Marinova E, Van Neer W, Waelkens M, Richards MP. Isotopic reconstruction of human diet and animal husbandry practices during the Classical-Hellenistic, Imperial and Byzantine Periods at Sagalassos, Turkey. American Journal of Physical Anthropology. 2012;149:157–71. doi: 10.1002/ajpa.22100 22729657

[108] Ma Y, Fuller BT, Wei D, Shi L, Zhang X, Hu Y, et al. Isotopic perspectives (δ13C, δ15N, δ34S) of diet, social complexity, and animal husbandry during the proto-shang period (ca. 2000–1600 BC) of China. American Journal of Physical Anthropology. 2016;160(3):433–45. doi: 10.1002/ajpa.22980 . 26972928

[109] Wang X, Fuller BT, Zhang P, Hu S, Hu Y, Shang X. Millet manuring as a driving force for the Late Neolithic agricultural expansion of north China. Scientific Reports. 2018;8(1):5552. doi: 10.1038/s41598-018-23315-4 pub.1101852976. 29615636 PMC5882897

[110] Schutkowski H, Ogden A, editors. Sidon of the plain, Sidon of the Sea—reflections on Middle Bronze Age diet in the Eastern Mediterranean. Archaeology and History in the Lebanon 34–35; 2011/12.

[111] Triantaphyllou S, Richards MP, Zerner C, Voutsaki S. Isotopic dietary reconstruction of humans from Middle Bronze Age Lerna, Argolid, Greece. Journal of Archaeological Science. 2008;35(11):3028–34. doi: 10.1016/j.jas.2008.06.018

[112] Ingvarsson-Sundstrom A, Richards MP, Voutsaki S. Stable isotope analysis of the Middle Helladic population from two cemeterues at Asine. Mediterranean Archaeology & Archaeometry. 2009;9:1–14.

[113] Petroutsa EI, Manolis SK. Reconstructing Late Bronze Age diet in mainland Greece using stable isotope analysis. Journal of Archaeological Science. 2010;37(3):614–20. doi: 10.1016/j.jas.2009.10.026

[114] Touzeau A, Amiot R, Blichert-Toft J, Flandrois J-P, Fourel F, Grossi V, et al. Diet of ancient Egyptians inferred from stable isotope systematics. Journal of Archaeological Science. 2014;46(0):114–24. doi: 10.1016/j.jas.2014.03.005

[115] Van Neer W, Fuller BT, Fahy GE, De Cupere B, Bouillon S, Uytterhoeven I, et al. Early Byzantine fish consumption and trade revealed by archaeoichthyology and isotopic analysis at Sagalassos, Turkey. Journal of Archaeological Science: Reports. 2024;53:104322. doi: 10.1016/j.jasrep.2023.104322

[116] Brozou A, Fuller BT, Grimes V, Van Biesen G, Ma Y, Boldsen JL, et al. Aquatic resource consumption at the Odense leprosarium: Advancing the limits of palaeodiet reconstruction with amino acid δ13C measurements. Journal of Archaeological Science. 2022;141:105578. doi: 10.1016/j.jas.2022.105578

[117] Fontanals-Coll M, Soncin S, Talbot HM, von Tersch M, Gibaja JF, Colonese AC, et al. Stable isotope analyses of amino acids reveal the importance of aquatic resources to Mediterranean coastal hunter–gatherers. Proceedings of the Royal Society B: Biological Sciences. 2023;290(1993):20221330. doi: 10.1098/rspb.2022.1330 36809804 PMC9943639

[118] Issar AS, Zohar M, editors. Climate change: environment and history of the Near East2007.

[119] Langgut D, Finkelstein I, Litt T. Climate and the Late Bronze Collapse: New Evidence from the Southern Levant. Tel Aviv. 2013;40(2):149–75. doi: 10.1179/033443513X13753505864205

[120] Finkelstein I, Harald Neumann F, Langgut D, Litt T, Stein M. Vegetation and Climate Changes during the Bronze and Iron Ages (∼3600–600 BCE) in the Southern Levant Based on Palynological Records. Radiocarbon. 2015;57(2):217–35. Epub 2016/02/09. doi: 10.2458/azu_rc.57.18555

[121] Coombes PMV, Barber KE. Environmental determinism in Holocene research: causality or coincidence? Area. 2005;37:303–11.

[122] Wright HEJ. Environmental Determinism in Near Eastern Prehistory. Current Anthropology. 1993;34:458–69.

[123] Yoffee N, Mcanany PA, editors. Collapse in Ancient Mesopotamia: What Happened, What Didn’t2009.

[124] Weiss H. The Northern Levant During the Intermediate Bronze Age: Altered Trajectories. In: Killebrew AE, Steiner M, editors. The Oxford Handbook of the Archaeology of the Levant: c 8000–332 BCE: Oxford University Press; 2013. p. 0.

[125] Brown AG, Carpenter RG, Walling DE. Monitoring fluvial pollen transport, its relationship to catchment vegetation and implications for palaeoenvironmental studies. Review of Palaeobotany and Palynology. 2007;147(1):60–76. doi: 10.1016/j.revpalbo.2007.06.005

[126] Marston JM. Archaeological markers of agricultural risk management. Journal of Anthropological Archaeology. 2011;30(2):190–205. doi: 10.1016/j.jaa.2011.01.002

[127] Riehl S. Variability in ancient Near Eastern environmental and agricultural development. Journal of Arid Environments. 2012;86:113–21. doi: 10.1016/j.jaridenv.2011.09.014

[128] Riehl S. Regional Environments and Human Perception: The Two Most Important Variables in Adaptation to Climate Change. 2017. p. 237–60.

[129] Riehl S. Significance of prehistoric weed floras for the reconstruction of relations between environment and crop husbandry practices in the near east. 2014. p. 135–52.

[130] Kamlah J, Riehl S. Agriculture in the Bronze Age Levant. A Companion to Ancient Agriculture2020. p. 193–209.

[131] Hazell CJ, Pound MJ, Hocking EP. High-resolution Bronze Age palaeoenvironmental change in the Eastern Mediterranean: exploring the links between climate and societies. Palynology. 2022;46:1–20.

[132] Millek JM, editor Exchange, Destruction, and a Transitioning Society. Interregional Exchange in the Southern Levant from the Late Bronze Age to the Iron I2020.

[133] Lawrence D, Philip G, Hunt H, Snape-Kennedy L, Wilkinson TJ. Long Term Population, City Size and Climate Trends in the Fertile Crescent: A First Approximation. PLOS ONE. 2016;11(3):e0152563. doi: 10.1371/journal.pone.0152563 27018998 PMC4809582

[134] Palmisano A, Lawrence D, de Gruchy MW, Bevan A, Shennan S. Holocene regional population dynamics and climatic trends in the Near East: A first comparison using archaeo-demographic proxies. Quaternary Science Reviews. 2021;252:106739. doi: 10.1016/j.quascirev.2020.106739

[135] Cruz-Silva E, Harrison SP, Prentice IC, Marinova E. Holocene vegetation dynamics of the Eastern Mediterranean region: Old controversies addressed by a new analysis. Journal of Biogeography. 2023;51:294–310.

