# Supplementary material for: Agropastoral and dietary practices of the northern Levant facing Late Holocene climate and environmental change: Isotopic analysis of plants, animals and humans from Bronze to Iron Age Tell Tweini
Source: PLoS One. 2024 Jun 12;19(6):e0301775. doi: 10.1371/journal.pone.0301775 (PMC11168695; doi:10.1371/journal.pone.0301775)
Supplement: S1 Checklist — (DOCX) [file pone.0301775.s001.docx]

Inclusivity in global research

PLOS’ policy on inclusivity in global research aims to improve transparency in the reporting of research performed outside of researchers’ own country or community and ensures that PLOS publications reporting global research adhere to high standards for research ethics and authorship. Authors of relevant research articles may be asked to complete the questionnaire below, which outlines ethical, cultural, and scientific considerations specific to inclusivity in global research. This questionnaire may be requested when researchers have travelled to a different country to conduct research, if research uses samples collected in another country, research with Indigenous populations or their lands, or if research is on cultural artefacts. Researchers travelling to another country solely to use laboratory equipment will not normally be required to complete the questionnaire. However, the questionnaire can be requested at the journal’s discretion for any submission – if you have been requested to complete this questionnaire by the PLOS journal you submitted to, please do so.

Please complete the questionnaire below and include this as a Supporting Information file with your manuscript. Note that if your paper is accepted for publication, this checklist will be published with your article in the supporting information files. Please ensure that you reference the checklist in the main body of your manuscript. We suggest adding a subsection ‘Inclusivity in global research’ to your Methods section and adding the following sentence: “Additional information regarding the ethical, cultural, and scientific considerations specific to inclusivity in global research is included in the Supporting Information (SX Checklist)”

The questions have been designed to be applicable to a wide range of study types, and there are subsections for both human subjects research and non-human subjects research. If any of the questions are not relevant to your research please mark them as “N/A” as appropriate.

**Ethical considerations, permits and authorship**

*This section is applicable to all research types.*

Provide details as to who granted permissions and/or consent for the study to take place in the Methods section of your manuscript. This should include the names of **all** ethics boards, governmental organizations, community leaders or other bodies that provided approval for the study. If individuals provided approval refer to these people by their role or title but do not list their name(s).

Reported on page number: page 1: Syrian Directorate-General of Antiquities and Museums, Damascus, Syria.

If there were any deviations from the study protocol after approval was obtained please provide details of these changes in the Methods section of your manuscript.
Did this study involve local collaborators that are residents of the country where the research was conducted or members of the community studied? If you do not have any authors from said communities, please provide an explanation for this below.

Reported on page number: N/A

The isotopical studies imply a great specialization and there is no local expert or academic in the region of Tell Tweini/Syria.

Everyone listed as an author should meet PLOS’ criteria for authorship and all individuals who meet these criteria should be included in the author byline, rather than the acknowledgements. Authorship criteria is based on the International Committee of Medical Journal Editors (ICMJE) Uniform Requirements for Manuscripts Submitted to Biomedical Journals - for further information please see here: <https://journals.plos.org/plosone/s/authorship>.

**Human subjects research (e.g. health research, medical research, cross-cultural psychology)**

Did you obtain written informed consent from a representative of the local community or region before the research took place? How did you establish who speaks for the community? Details of written informed consent obtained from study participants should be reported separately in the Methods section of your manuscript.

Our research is granted by the Syrian Directorate-General of Antiquities and Museums and all the aspects of our research with the local authorities of the Syrian Directorate-General of Antiquities and Museums successively headed by Directors-General, Dr. Bassam Jamous and Dr. Maamoun Abdulkarim.

How did members of the local community provide input on the aims of the research investigation, its methodology, and its anticipated outcome(s)?

Unfortunately, the local community does not have sufficient academic expertise on this subject.

When engaging with the local community, how did you ensure that the informed consent documents and other materials could be understood by local stakeholders?

The local community has been witness of the archaeological excavations in the site of Tell Tweini and they recognise the authority of the Syrian Directorate-General of Antiquities and Museums over the area.

Will the findings of the research be made available in an understandable format to stakeholders in the community where the study was conducted (e.g. via a presentation, summary report, copies of publications, etc.)? Please provide details of how this will be achieved.

The excavations and discoveries at Tell Tweini are open to the public and are elucidated through informative boards. Noteworthy findings are showcased at the archaeological museum of Jebleh/Syria.

**Non-human subjects research using specimens/ animals collected as part of the study, or those housed in archival collections. Examples include archaeology, paleontology, botany and zoology.**

Did the permission you obtained from a local authority to perform the study include an agreement on access to outputs and benefit sharing? This may include procedures to enable fair distribution of the benefits and resources arising from the research performed. Please include any details of Prior Informed Consent and Benefit Sharing Agreements obtained. These may be required by field-specific regulations, for example the Convention on Biological Diversity (CBD) and the associated Nagoya Protocol.

There is no clause in the permit granted by the Syrian Directorate-General of Antiquities and Museums that focuses on the aspects mentioned in the question.

If the material used in your study was imported, please A) provide the year it was imported and B) indicate whether permits were obtained to import/export the materials used, C) provide details of any permits obtained. If this information is not available, please indicate this.

All necessary permits were obtained for the described study (from the Syrian Directorate-General of Antiquities and Museums between 1999-2010), which complied with all relevant regulations.

If you used archival specimens, please state how the material used in your study was acquired by the institute it is held in and provide details of any permits obtained for the original excavations/ sample collection. If this information is not available, please indicate this.

N/A

How was the potential cultural significance of the materials collected in your study to local communities considered in your research design? Were Indigenous peoples and/or local researchers and institutions involved with archaeological excavations / collection of specimens? If so, please provide a description of their involvement.

Our research project is interdisciplinary. One of the main lines of research is the reconstruction of History, which is why, when our project was designed, the main objectives were to understand the material context of past societies in order to transmit new perspectives not only to local communities, but to everyone.

If your manuscript includes photographs of human remains please indicate whether authors obtained permission from descendants or affiliated cultural communities to do so.

N/A
