# Supplementary material for: Agropastoral and dietary practices of the northern Levant facing Late Holocene climate and environmental change: Isotopic analysis of plants, animals and humans from Bronze to Iron Age Tell Tweini
Source: PLoS One. 2024 Jun 12;19(6):e0301775. doi: 10.1371/journal.pone.0301775 (PMC11168695; doi:10.1371/journal.pone.0301775)
Supplement: S1 File — (DOCX) [file pone.0301775.s002.docx]

**Agropastoral and dietary practices of the northern Levant facing Late Holocene climate and environmental change: Isotopic analysis of plants, animals and humans from Bronze to Iron Age Tell Tweini**

Benjamin T. Fuller^1‡*^, Simone Riehl^2‡*^, Veerle Linseele^1,3^, Elena Marinova^1,3^, Bea De Cupere^3^, Joachim Bretschneider^4^, Michael P. Richards^5§^, Wim Van Neer^1,3§^

*^1^Laboratory of Biodiversity and Evolutionary Genomics, Centre for Archaeological Sciences, University of Leuven, Ch. Debériotstraat 32, B-3000 Leuven, Belgium*

*^2^Institute of Archaeological Science, University of Tübingen and Senckenberg Center for Human Evolution and Palaeoenvironment (HEP), Hölderlinstraße 12, D-72070 Tübingen Germany*

*^3^Royal Belgian Institute of Natural Sciences, Vautierstraat 29, B-1000 Brussels, Belgium*

*^4^Department of Archaeology, Ghent University, Sint-Pietersnieuwstraat 35, B-9000 Ghent, Belgium*

*^5^Department of Archaeology, Simon Fraser University, Burnaby, British Columbia V5W 1S6 Canada*

*Address for correspondence:

Email: benjamin.fuller@sthughs-oxford.com

Email: simone.riehl@uni-tuebingen.de

^‡^ = Equal first authors

§ = Equal last authors

**Supplementary Figures:**

**
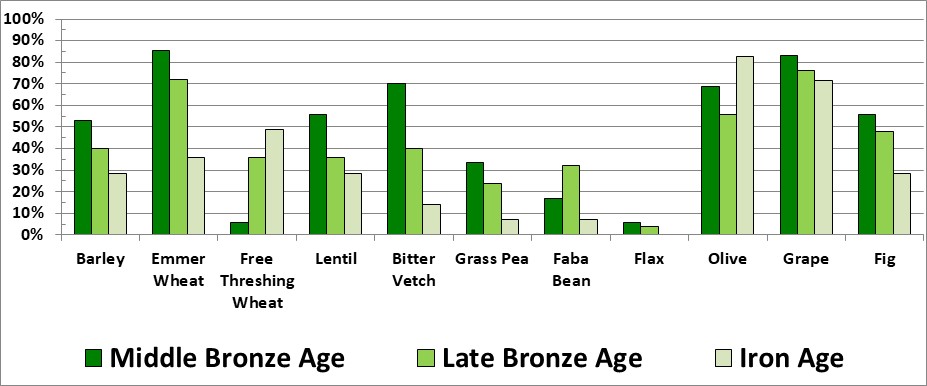
**

Figure S1. Ubiquity (occurrence) of the main crop plants through the study periods. (Note - number of samples for each period given in Figure S2). For the Early Bronze Age, it is not possible to calculate ubiquity as there is only one sample and the ubiquity is based on the percentage value of samples in which a taxon occurs from the total number samples per period. It is usually calculated when 10 or more samples were studied from a period [1].


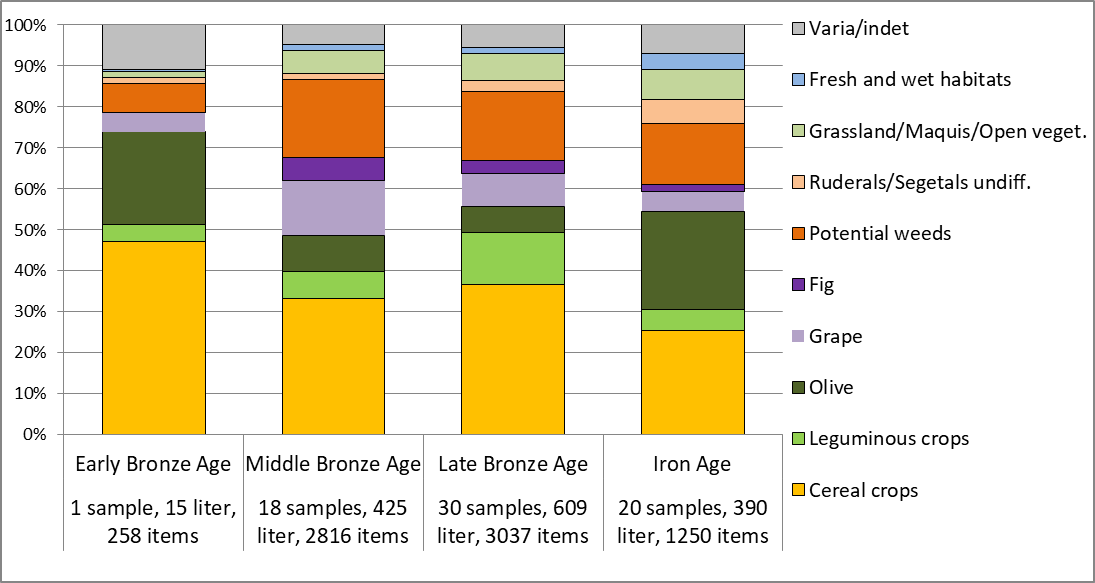


Figure S2. Percentage proportions of the different cultivated and natural habitats as represented in the archaeobotanical samples at Tell Tweini [2].


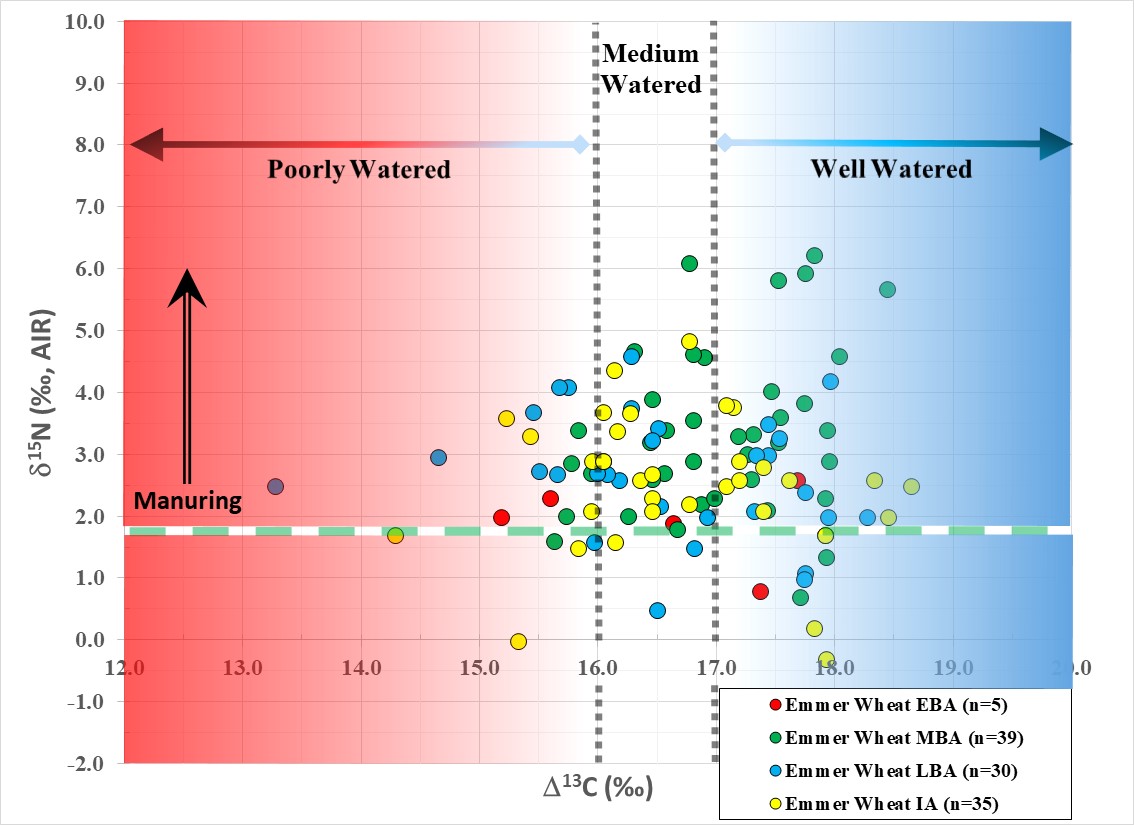


Figure S3. Emmer wheat Δ^13^C and δ^15^N values plotted by time period. Horizontal green line denotes the upper error range δ^15^N value of the estimated natural forage (1.8‰) for all time periods [3]. Emmer wheat δ^15^N results >1.8‰ potentially indicate the addition of manure as a fertilizer. Vertical black dotted lines denote the Δ^13^C water status boundaries [4,5].


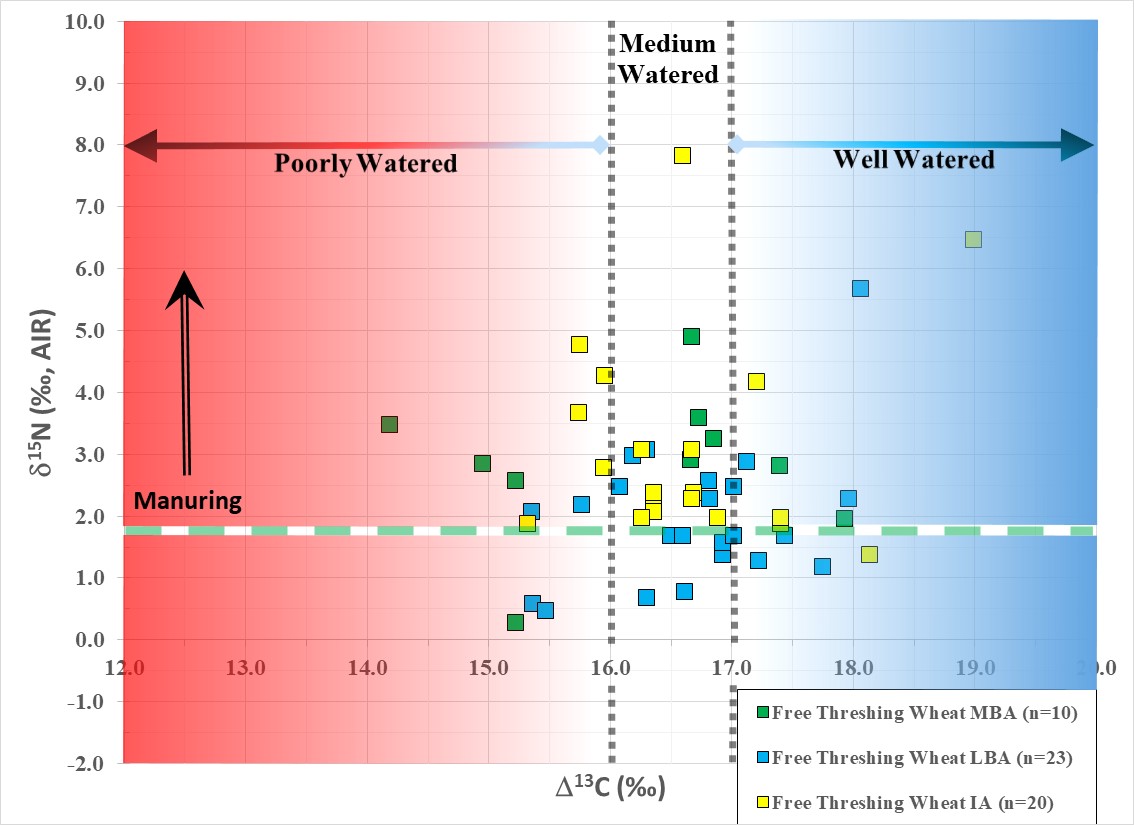


Figure S4. Free-threshing wheat Δ^13^C and δ^15^N values plotted by time period. Horizontal green line denotes the upper error range δ^15^N value of the estimated natural forage (1.8‰) for all time periods [3]. Free-threshing wheat δ^15^N results >1.8‰ potentially indicate the addition of manure as a fertilizer. Vertical black dotted lines denote the Δ^13^C water status boundaries [4,5].


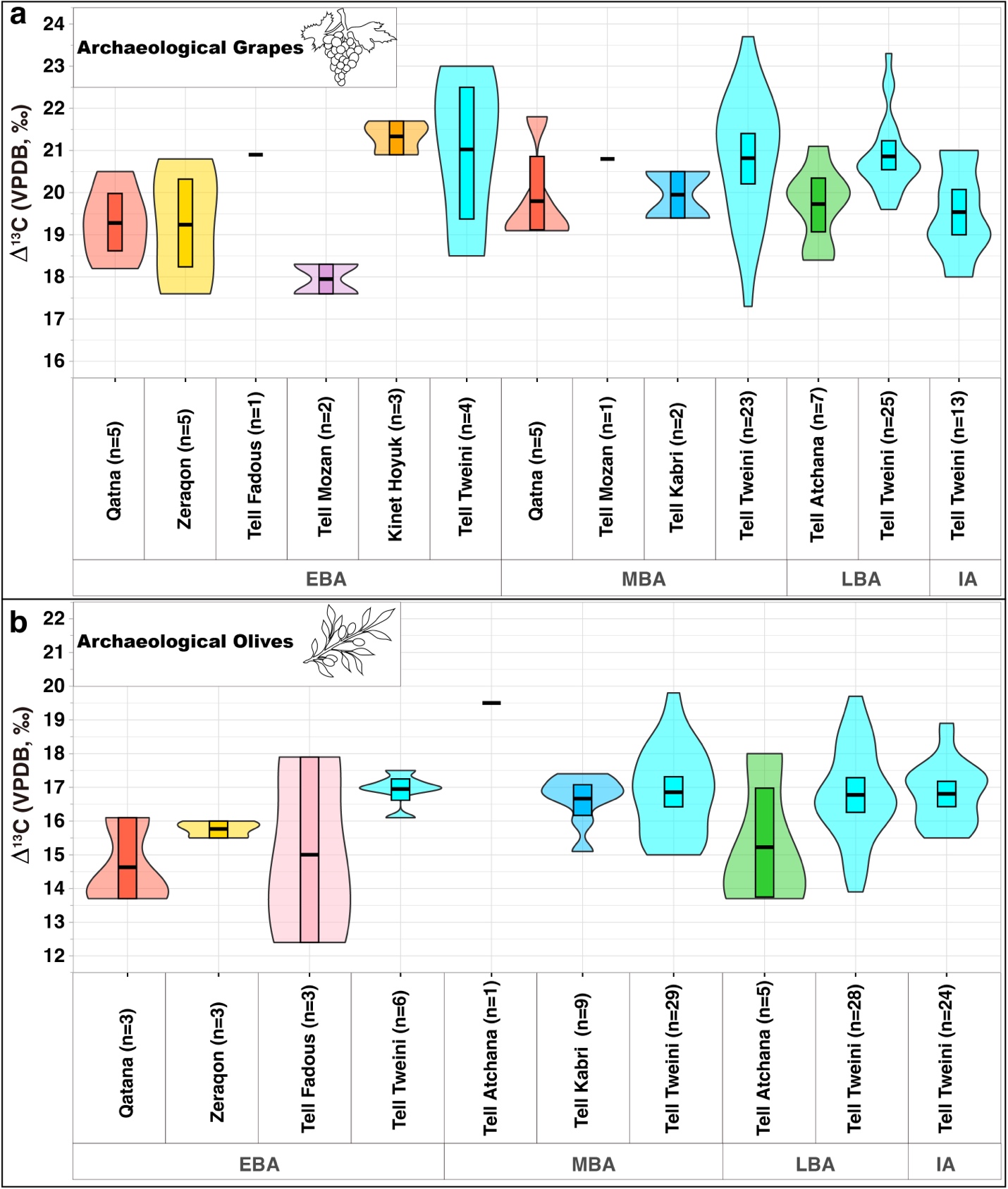


Figure S5. Violin plots of (a) grape and (b) olive Δ^13^C results from different archaeological sites in the Near East. All data, except for Tel Kabri [6] are from Riehl, unpublished. Background archaeobotanical results are available for Qatna [7], Zeraqon [8], Tell Fadous [9], Tell Mozan [10], and Tell Atchana [11]. Boxes in the middle of the violins represent the mean Δ^13^C and its 95% confidence intervals for each group (Plots generated using RStudio V.1.4.1717 and final layout created using Adobe Illustrator CC 2019 V.23.1.1).


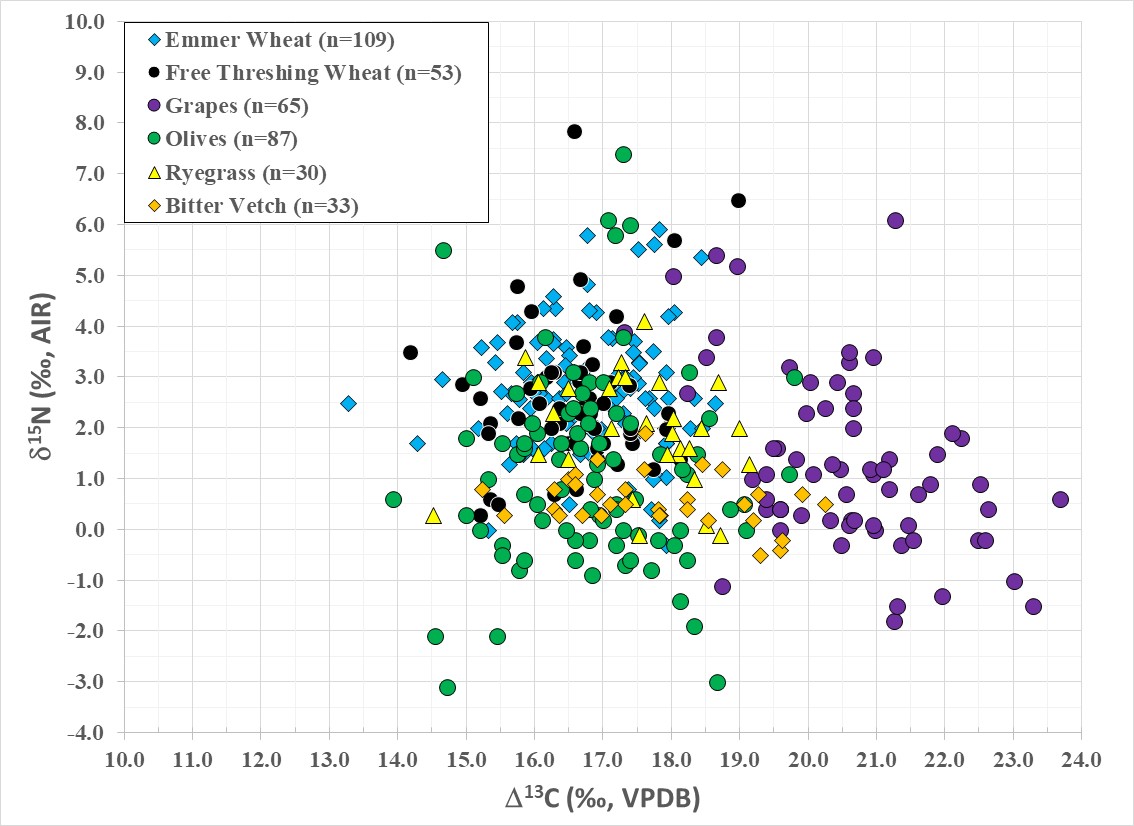


Figure S6. All plant isotopic δ^15^N vs Δ^13^C results for Tell Tweini.


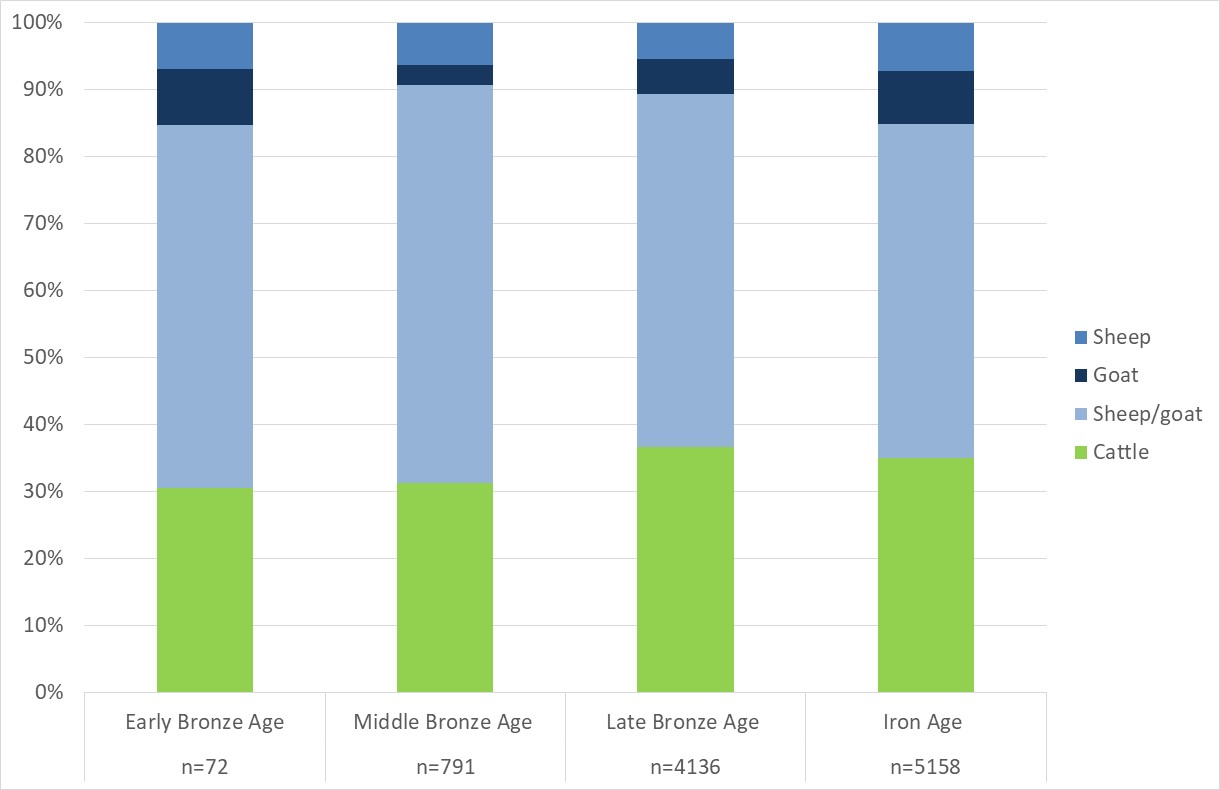


Figure S7. Summary of the relative importance of domestic cattle and sheep/goats by phase at Tell Tweini (n = number of identified specimens) [2].

**References**

[1] Pearsall, D. M. (2015). Paleoethnobotany: A handbook of procedures. (Third edition).

Walnut Creek, California: Left Coast Press.

[2] Linseele V, Marinova E, De Cupere B, van der Valk J, Vandorpe P, Van Neer W. Bronze and Iron Age Palaeo-Economy in a Changing Environment. The Bioarchaeology of Tell Tweini, on the Northern Levantine Coast. . In: Bretschneider J, Jans G, editors. About Tell Tweini (Syria): Artefacts, Ecofacts and Landscape. Research Results of the Belgian Mission, Orientalia Lovaniensia Analecta Series 281. Leuven: Peeters publishers; 2019. p. 417-617.

[3] Nitsch E, Andreou S, Creuzieux A, Gardeisen A, Halstead P, Isaakidou V, et al. A bottom-up view of food surplus: using stable carbon and nitrogen isotope analysis to investigate agricultural strategies and diet at Bronze Age Archontiko and Thessaloniki Toumba, northern Greece. World Archaeology. 2017;49(1):105-37. doi: 10.1080/00438243.2016.1271745.

[4] Wallace M, Jones G, Charles M, Fraser R, Halstead P, Heaton THE, et al. Stable carbon isotope analysis as a direct means of inferring crop water status and water management practices. World Archaeology. 2013;45(3):388-409. doi: 10.1080/00438243.2013.821671.

[5] Wallace MP, Jones G, Charles M, Fraser R, Heaton THE, Bogaard A. Stable Carbon Isotope Evidence for Neolithic and Bronze Age Crop Water Management in the Eastern Mediterranean and Southwest Asia. PloS one. 2015;10(6):e0127085-e. doi: 10.1371/journal.pone.0127085. PubMed PMID: 26061494.

[6] Riehl S. Chapter 25 Stable Isotope Measurements on Seed Remains. In: Cline EH, Ratzlaff A, editors. Excavations at Tel Kabri III. Leiden, Niederlande: Brill; 2023. p. 318-27. [doi.org/10.1163/9789004548336_026](https://doi.org/10.1163/9789004548336_026)

[7] Riehl S. Plant production at Qatna in the environmental and supra-regional economic context. In: Pfälzner P, editor. Qatna and the networks of Bronze Age globalism Akten einer internationalen Konferenz in Stuttgart im Oktober 2009. Qatna Studien Vol. 8, 2015. p. 477-487.

[8] Riehl S. Archaeobotany at the Early Bronze Age settlement of Hirbet ez-Zeraqon: a preliminary report. Zeitschrift des Deutschen Palästina-Vereins. 2004;120(2):102-22.

[9] Riehl S. Flourishing surplus economy in coastal lands: Archaeobotany and stable carbon isotopes. In: Genz H, editor. Tell Fadous-Kfarabida I: the site and its environment. 2016. 52 p.

[10] Riehl S. Plant production in a changing environment – The archaeobotanical remains from Tell Mozan. In: Deckers K, Doll M, Pfälzner P, Riehl S, editors. Ausgrabungen 1998 - 2001 in der Zentralen Oberstadt von Tall Mozan / Urkeš: The Development of the Environment, Subsistence and Settlement of the City of Urkeš and its Region. SUN, Serie A, Vol. 3.2010. p. 13-158.

[11] Riehl S. Flourishing agriculture in times of political instability – the archaeobotanical and isotopic evidence from Tell Atchana. In: Yener KA, editor. Excavations in the plain of Antioch Tell Atchana, Ancient Alalakh, a Bronze Age capital in the Amuq Valley, Turkey The 2003-2004 excavation seasons. Istanbul: Koç University Press; 2010. p. 123-36.
